# Unraveling the Effects of Selection and Demography on Immune Gene Variation in Free-Ranging Plains Zebra (*Equus quagga*) Populations

**DOI:** 10.1371/journal.pone.0050971

**Published:** 2012-12-14

**Authors:** Pauline L. Kamath, Wayne M. Getz

**Affiliations:** 1 Department of Environmental Science, Policy, and Management, University of California, Berkeley, California, United States of America; 2 School of Mathematical Sciences, University of KwaZulu-Natal, Durban, South Africa; University of Massachusetts, United States of America

## Abstract

Demography, migration and natural selection are predominant processes affecting the distribution of genetic variation among natural populations. Many studies use neutral genetic markers to make inferences about population history. However, the investigation of functional coding loci, which directly reflect fitness, is critical to our understanding of species' ecology and evolution. Immune genes, such as those of the Major Histocompatibility Complex (MHC), play an important role in pathogen recognition and provide a potent model system for studying selection. We contrasted diversity patterns of neutral data with MHC loci, ELA*-DRA* and *-DQA*, in two southern African plains zebra (*Equus quagga*) populations: Etosha National Park, Namibia, and Kruger National Park, South Africa. Results from neutrality tests, along with observations of elevated diversity and low differentiation across populations, supported previous genus-level evidence for balancing selection at these loci. Despite being low, MHC divergence across populations was significant and may be attributed to drift effects typical of geographically separated populations experiencing little to no gene flow, or alternatively to shifting allele frequency distributions driven by spatially variable and fluctuating pathogen communities. At the *DRA*, zebra exhibited geographic differentiation concordant with microsatellites and reduced levels of diversity in Etosha due to highly skewed allele frequencies that could not be explained by demography, suggestive of spatially heterogeneous selection and local adaptation. This study highlights the complexity in which selection affects immune gene diversity and warrants the need for further research on the ecological mechanisms shaping patterns of adaptive variation among natural populations.

## Introduction

Spatiotemporal fluctuations in pathogens generate complex patterns in the distribution of gene variation among host populations. Pathogens affect hosts not only by driving changes in population size, but also by eliciting an evolutionary response in host immune genes. Stochastic demographic factors (e.g. chance events associated with births, deaths and movement) influence, in the same way, rates of gene flow and genetic drift at all loci in an organism's genome [1], [2]. Thus, neutral genetic data is informative for studying demography [3], for example, to examine relatedness, mating behavior, dispersal patterns, changes in population size, population genetic structure and speciation [4]–[6]. It is functional gene variation, however, that reflects natural selection, fitness and the potential to adapt to changing environments, making it of critical importance to the study of evolution, ecology and conservation [7], [8]. Pathogen-mediated selection is expected to affect specific genomic regions, depending on gene function and the magnitude of the fitness consequence to the host. Contrasting patterns of diversity at neutral and immune response loci can be particularly informative for understanding how hosts adapt to pathogens, by illuminating the relative effects of selection versus demography on gene variation and population dynamics [9]–[14].

The Major Histocompatibility Complex (MHC) is a family of genes that plays a critical role in vertebrate immune function. Class II MHC genes encode cell-surface glycoproteins, with peptide binding regions (PBRs) that are responsible for recognizing foreign antigens from extracellular pathogens (e.g. eukaryotic parasites and bacteria) and presenting them to helper T-lymphocytes to elicit an immune response. These genes typically exhibit elevated levels of polymorphism and highly divergent variants have been shown to persist over long time periods, even through the course of speciation events [15], a phenomenon largely explained by balancing selection [16]. Variation in the MHC has been shown to be concentrated within the PBR [17], suggesting that pathogen recognition is a selective driving mechanism, with increased allelic diversity allowing for recognition of a broader spectrum of pathogens [18]. Beyond implications for modulating disease resistance, MHC variants are also known to influence other biological traits such as mate preference, kin recognition and maternal-fetal interactions (reviewed in [19]–[21]). As a well-studied system, the MHC remains an important model with which to test hypotheses regarding the influence of selection on host genetic diversity.

The mechanism by which pathogen-mediated balancing selection maintains diversity in MHC genes has been extensively debated in the literature [8], [18], [20]–[24]. At present, there are three primary hypotheses that have been widely discussed: (*i*) overdominant selection [25], (*ii*) frequency-dependent selection [26] and (*iii*) fluctuating selection [27], [28]. The theory of overdominant selection (or heterozygote advantage) makes the assumption that heterozygous individuals recognize a broader range of pathogens and therefore have a fitness advantage over homozygotes [25]. Whereas, under frequency-dependent selection (or rare-allele advantage) the advantage of a particular allele is suspected to vary with its frequency, such that as pathogens evolve to evade the more common host alleles, rare alleles may become advantageous by conferring host resistance [29], [30]. In reality, however, natural populations are exposed to fluctuating environmental conditions and, subsequently, host-pathogen interactions are similarly expected to vary spatiotemporally [28]. Empirical genetic evidence for geographic heterogeneity in selective pressures has been shown in fish [10], birds [31], [32] and mammals [33], [34]. A selection model demonstrated that temporal variation in pathogen resistance may be sufficient to maintain polymorphism in the absence of both heterozygote and rare-allele advantage [35]. While it is generally accepted that any or all of these proposed mechanisms can play a role in shaping the distribution of MHC variation, distinguishing between them in natural populations can be challenging due to similarities in the expected genetic outcomes [23].

Plains zebra (*Equus quagga*) experience spatiotemporal variability in pathogen pressures across localities (Table 1). In Etosha National Park, Namibia, *E. quagga* are the main host species of anthrax, a deadly disease caused by the gram-positive bacterium, *Bacillus anthracis*, which occurs in severe and consistent annual outbreaks [36], [37]. In contrast, the zebra population of Kruger National Park, South Africa, suffers from sporadic and less intensive anthrax infections, with outbreaks occurring on an approximate decadal cycle [38]. MHC genes are known to be involved in immune response to bacterial pathogens, and associations have been documented between polymorphisms in classical human leukocyte antigen haplotypes (HLA-DR-DQ) and heterogeneity in host immunoglobulin G antibody response to the *B. anthracis* protective antigen following administration of the anthrax vaccine [39].

**Table 1:** Summary of differences in parasitism between *E. quagga* populations.

	Etosha	Kruger	Literature Source
*No. Nematoda spp.*	21	32	[41]
*No. Strongylinae spp.*	1	6	[41]
*Nematode prevalence*	>98%	100%	[40], [112]
*No. Ixodidae tick spp.*	4	7	[42], [43]
*Anthrax seasonality*	Wet season	Dry season	[36]–[38], [113]
*Anthrax frequency*	Annual	Periodic	[36]–[38]
*Main species affected*	Plains zebra	Greater kudu	[36], [38], [113]

Parasitism characterized by population (Etosha *vs.* Kruger) in terms of the number of documented nematode species from the phylum Nematoda and the Subfamily Strongylinae, prevalence of strongyle nematodes (Order: Strongylida), number of tick species (Family: Ixodidae), seasonality of anthrax outbreaks in plains ungulates, approximate frequency of anthrax outbreaks, and the main host species affected by anthrax in terms of the highest number of recorded anthrax mortalities.

Other than anthrax, zebra in Etosha are appreciably infected by gastrointestinal (GI) parasites, found in the population at nearly 100% prevalence [40]. Gastrointestinal nematode prevalence is similarly high in the Kruger population, but there is a greater nematode species richness than in Etosha [41]. Differences in parasite species richness between these populations is also apparent with regards to the ectoparasite community, with higher Ixodidae tick species diversity in Kruger [42] than in Etosha [43]. Recent studies have provided evidence for balancing selection acting on two MHC (or Equine Lymphocyte Antigen; ELA) genes, *DRA* and *DQA*, over the history of the genus *Equus*, as well as positive selection acting at sites responsible for binding foreign peptides [44], [45]. MHC variation has been explored in a captive population of *E. przewalski* [46], however no studies, to our knowledge, have examined the evolution of MHC genes in wild equid populations. The differential pathogen pressures in Etosha and Kruger provide an excellent natural system for examining the effects of selection and demography on MHC genes.

Contrasting the levels and distribution of genetic diversity at MHC genes with those expected from neutral expectations within and among populations has been frequently employed to elucidate the effects of selection on MHC genes [9]–[11], [14], [32]. In this study, we examined the distribution of genetic variation in the ELA- *DRA* and *DQA* PBR within zebra populations differing in parasite richness and severity (Table 1) to shed light on how selection may affect MHC gene variation. A baseline for demography, including genetic structure and inferences regarding changes in population size, was established through analyses combining the use of neutral microsatellite and nuclear intron data. We employed tests for selection and demography on both functional and neutral data to reveal differences in selective pressures occurring across loci and zebra populations. We expect to discover a signature of balancing selection in ELA genes across zebra populations, given their functional role in immune response. We also predict to find evidence of heterogeneous selection pressures acting across populations, given the differences in parasite species richness in these two localities. These results provide valuable insight for understanding the importance of immune genes in local adaptation and for identifying candidate alleles that may play an important role in pathogen resistance.

## Materials and Methods

### Ethics statement

This study was approved by the Namibian Ministry of Environment and Tourism (Permit #1220/2007) and South African National Parks, South Africa (Contract, Project title: “The role of host genetics in susceptibility to anthrax among Burchell's zebra of southern Africa”). All field sampling was conducted in accordance to the requirements and guidelines outlined. The sample collection protocol was also approved by the Animal Care and Use Committee (Protocol #R217-0510B) at the University of California, Berkeley.

### Study populations and sample collection

Blood, tissue and fecal samples were collected from two *E. quagga* populations of southern Africa: Etosha National Park, Namibia (*n* = 84), and Kruger National Park, South Africa (*n* = 89). Fecal samples were collected immediately following defecation and could be attributed to individual zebra. Sample preservation and genomic DNA extraction protocols are outlined in Kamath & Getz [45]. DNA extracted from fecal material occasionally resulted in failed PCR-amplifications and was attributed to enzyme degradation, hydrolytic and oxidative damage. Therefore, we often used only subset of the data in downstream genetic analyses (between 41–71% of samples were successfully typed per locus; Table S1).

From 2000 through 2012, Etosha zebra population abundance was estimated from aerial survey data as being relatively stable, fluctuating between approximately 13,000 to 16,000 individuals [Namibian Ministry of Environment and Tourism, unpublished data]. In Kruger, zebra population surveys from 1980 to 1993 similarly indicate that abundance was stable, ranging between 21,454 to 33,164 individuals per year, estimates that may only represent 60–85% of the true population size [47], [48].

### ELA amplification, cloning and sequencing

The exon 2 coding regions of the ELA*-DRA* and *-DQA* were amplified from genomic DNA following PCR and sequencing reaction protocols described in Kamath & Getz [45]. Amplified fragments were 246 and 205 bp and encompassed the PBR of the *DRA* and *DQA*, respectively. We sequenced loci in forward and reverse directions to confirm heterozygous base positions. Sequence chromatograms were edited and aligned manually using Geneious 5.0 [49]. Allelic phase for *DRA* heterozygotes was determined with the haplotype reconstruction program, PHASE v2.1 [50], which has been shown to perform well, even with MHC sequences comprised of many heterozygous sites [51]. We conducted two independent runs with different initial random seeds and upheld a threshold posterior probability of 0.80 for haplotype confirmation, which is higher than the suggested cutoff [52]. For all but two individuals that were included in further analyses, haplotype posterior probabilities fell between 0.95 and 1.

The *DQA* locus is known to be highly variable and our data may represent alleles from two loci [45], [53]. Although our primers appeared to preferentially amplify only one locus, for the sake of simplicity we herein refer to all *DQA* sequences as ‘alleles’ despite the possibility they may come from the different loci. To identify alleles in heterozygous individuals, molecular cloning was carried out using a TOPO-TA® cloning kit with Mach 1™ T1R competent cells (Invitrogen). We minimized polymerase errors, heteroduplex and chimera formation by using a high fidelity polymerase (Platinum® Taq DNA Polymerase High Fidelity, Invitrogen) in the initial PCR. Amplicons were run on a 1% agarose gel and visualized using GelStar® Nucleic Acid Gel Stain (Cambrex Bio Science Rockland, Inc.) and the Dark Reader transilluminator system (Clare Chemical) which does not damage DNA and also increases the transformation efficiency during subsequent cloning procedures. Bands were excised and purified using QIAquick Gel Extraction Kits (Qiagen), ligated into pCR®4 TOPO vectors and transformed into *E. coli* competent cells. Following an overnight incubation at 37°C, sixteen to twenty-three positive clones were picked per individual and clones were directly sequenced using the same primers as in the initial PCR. Alleles were confirmed with at least two observations (i.e. amplified in at least two independent PCRs from the same individual or seen in two different individuals). Sequences not meeting these criteria were considered to be erroneous and not considered in subsequent analyses.

### *β-Fibrinogen* intron amplification

The *Fibrinogen* beta chain gene, intron 7 (*β-Fibr*) appears to be evolving neutrally and has been demonstrated to be an informative marker for systematic and evolutionary studies in both birds and mammals [54]–[56]. We amplified 668 bp of *β-Fibr* with primers developed using the publicly-available horse genome and Primer3 [57]: *β-Fibr10* (5′-CAGTAGTATCTGCCGTTTGG-3′) and *β-Fibr11* (5′-GAGGGCGACAAATACCAAC- 3′) (Protocol S1). Amplified products were cleaned using Exo-SAP-IT (USB Corporation) and sequenced in both directions on an ABI 3730 sequencer (Applied Biosystems). Cycling conditions were followed as described for ELA loci and haplotype determined using PHASE v2.1 [50]. Due to the presence of a length polymorphism, we used the program OLFinder [58] to resolve heterozygous genotypes when an insertion/deletion (indel) event was evident.

### Microsatellite genotyping

We genotyped 15 microsatellite loci previously isolated from the horse (*Equus callabus*) (Protocol S1, Table S2). Forward primers were modified at the 5′-end by the addition of a fluorescent label: HEX, 6-FAM (Invitrogen), NED or PET (Applied Biosystems). Allele fragments were scored for size against the LIZ-500 size standard through electrophoresis using an ABI3730 DNA Analyser, followed by visualization with GeneMapper v.4.0 (Applied Biosystems) software.

A portion of the samples were derived from feces, and thus were expected to be subject to high genotyping error rates and allelic dropout. To account for these issues, we used a comparative genotyping protocol [59] (Figure S1), as a modification of the multi-tubes approach [60]. This protocol has been shown to efficiently reduce error rates by minimizing the number of PCRs necessary to arrive at a consensus genotype [61]. Furthermore, we characterized and quantified genotyping error using paired blood and fecal samples (*n* = 42) obtained from zebras in Etosha.

Fisher's exact tests for Hardy-Weinberg (H-W) equilibrium and genotypic linkage disequilibrium (LD) between pairs of loci were conducted in GENEPOP 4.0 [62] to test that microsatellite loci followed the assumptions of neutrality. Significance of exact tests was determined with a Markov chain algorithm [63] using default parameters, and corrected for multiple comparisons through a sequential Bonferroni procedure [64]. Null allele frequency (NAF) (frequency of non-amplified alleles resulting in an apparent homozygote) was estimated per locus (10,000 pseudoreplicates) using the expectation maximum algorithm implemented in FreeNA [65]. Two loci, Asb23 and Lex33, had low amplification success in samples from Kruger, with 63% and 71% missing data, respectively, and were excluded from all further analyses on both populations.

### Intra-population genetic diversity

General patterns of intra-population diversity were assessed by calculating the average number of alleles (*A*) and expected heterozygosity (*H*_E_) at all loci using GenAlEx 6.2 [66]. Allelic richness, being sensitive to differences in sample size, was corrected for by rarefaction (*A*_CORR_) in HP-RARE 1.1 [67]. Allelic variation in zebra populations was quantified at sequence-based loci (*β-Fibr*, *DRA*, *DQA*) in terms of number of segregating sites (*S*: [68]), haplotype diversity (*H*_D_: [68]), nucleotide diversity (*π*: [69]) and the mean number of pair-wise nucleotide differences (*k*: [70]) in DNAsp v5 [71]. Empirical distributions were generated to determine sampling variance and standard deviations of parameter estimates.

Given that the *DQA* sequences may be derived from two unresolved loci, we were not able to assign specific alleles to a locus. Therefore, we estimated allele frequencies as the number of individuals carrying a particular allele out of the total number of alleles (see [14], [32]). Homozygotes were assumed to have two copies of the observed allele, but in cloned heterozygotes (with ≥2 sequences identified) each allele observed was counted only once. We recognize that this method may underestimate the frequency of common alleles and likewise overestimate rare allele frequency. Therefore, we alternatively assessed intra- and inter-population variability at the *DQA* locus using measures independent of allele frequency: mean number of alleles per individual, total number of alleles per population and average percent difference (APD). APD was calculated based on the average percentage of sequences that differ among all possible individual pair-wise comparisons, as outlined in Yuhki and O'Brien [72], and can be used as a reliable measure of within-population genetic variation from multi-locus data [14]. To facilitate comparisons across loci, we similarly estimated APD for both the *DRA* and *β-Fibr* loci. As diversity data was not normally distributed, we used a non-parametric Kruskal-Wallis rank randomization test to test the null hypothesis of group-mean equality. Differences in variance between population samples were addressed by a Levene test based on absolute residuals of each observation to the respective population mean. A Wilcoxon sign-rank test (*Z* test) was performed to account for unequal variance between population samples in group-mean comparisons. In addition, we tested for differences in the distribution of *DQA* allele number per individual across populations through goodness-of-fit contingency analyses, with significance assessed through calculation of the Chi Square statistic (*χ^2^*). All statistical analyses were performed using JMP 4.0 (SAS Institute Inc.) software. As we found greater than two alleles in only a small number of individuals (*n* = 10), we also conducted all analyses excluding these individuals to confirm observed patterns in the data.

### Population structure and clustering

Selection may affect the distribution of mutations and frequency of alleles within and among populations. Therefore, comparing the population differentiation observed at functional and neutral loci may be informative for understanding the nature of selection [20], [21]. Under the effects of balancing selection, population divergence is expected to be lower at MHC loci relative to neutral loci, due to a more even spatial distribution of genetic variation, whereas if the mode of selection varies across populations, we may expect MHC divergence to be higher than estimates based on neutral data. We contrasted the partitioning of genetic variation at ELA loci to that of neutral data to control for the confounding effects of migration and population size, and shed light on selection heterogeneity across populations. Population differentiation was assessed in Arlequin v3.5 [73] by conventional *F*-statistics (*F*_ST_: [74]). The *F*_ST_ estimator, *Φ*_ST_ [75], was calculated for sequence data, using a Kimura-2 parameter distance matrix, with significance determined by 1,000 permutations. Ninety-five percent confidence intervals were generated for microsatellite *F*_ST_ estimates by bootstrapping (10,000 replicates) in FSTAT v2.9 [76] and ELA values falling outside this interval were considered to be significantly different [9]. ELA estimates were directly compared to *F*_ST_ values derived from the nuclear intron, *β-Fibr*. Population divergence was further assessed by global exact tests of differentiation (Markov chain steps = 100,000, dememorization steps = 10,000).

As the variability of a genetic marker increases, estimates of *F*_ST_ and its family of analogs have been shown to approach zero (i.e. equivalent to 100% genetic similarity), sometimes even among fully differentiated subpopulations [77]. This observation suggests these estimators may not be comparable across the markers and, therefore, may not represent ‘true’ estimates of the level of similarity among populations. To account for this, we calculated Jost's *D* actual differentiation estimator, *D*_est_ [78] in SMOGD [79]. For microsatellite-based estimates of *D*_est_, we reported the harmonic mean across all loci.

At the *DQA* locus, population allele frequencies used in *F*_ST_ computations are potentially biased due to overestimation of rare allele frequencies (as discussed previously). Therefore, we calculated a measure of differentiation *F*′ (analogous to *F*_ST_), to account for this bias in allele frequencies. This measure is derived from the similarity index [80] and because it uses percent similarity among pair-wise sequences of individuals to estimate population sub-division, it is analogous to APD. Standard errors of *F*′ were estimated by applying a Taylor expansion approximation [81]. Estimates of *F*′ were also estimated for the *DRA* and *β-Fibr* to validate comparisons of differentiation across loci.

Zebra population structure was further assessed using the Bayesian clustering algorithm in the program STRUCTURE v2.3 [82], with analyses conducted on neutral data (13 microsatellite loci and the *β-Fibr* intron), and both ELA loci independently. The genetic clustering algorithm maximizes Hardy-Weinberg proportions, under a model allowing for population admixture and the assumption that alleles were correlated. We considered models that specified *K* = 1 through 5 genetic clusters and conducted 10 independent runs for each value of *K*, each totaling 1,000,000 MCMC steps (burnin = 100,000). We inspected both the maximum log probability for a given model, Pr(X|K), and the rate of change in the log probability of the data between successive *K* values, Δ*K*, to determine the most probable number of genetic clusters given the dataset [83]. Results from the 10 replicate runs were summarized for the most probable *K* using the program CLUMPP v1.1 [84] and results were visualized using *Distruct* [85].

### Demographic inference

We constructed mismatch distributions using the *β-Fibr* sequence data in Arlequin 3.5 [73] to test the null hypothesis of recent increase in population size. Mismatch analyses compared the observed frequency distribution of pair-wise differences to the expected unimodal distribution of a population that has undergone a sudden expansion, generated through coalescent simulations [86], [87]. Multimodality and deviations from the expected distribution may be indicative of a stationary population at demographic equilibrium. Goodness-of-fit tests for population expansion were conducted by calculating the sum of squared deviation (SSD) and raggedness index (RI) [88], and significance determined by 10,000 coalescent simulations.

Historical changes in zebra population sizes were characterized with a coalescent-based model in LAMARC v2.1.5 [89] using microsatellite data. We conducted two independent Bayesian runs under the Brownian motion approximation mutation model [90], with a sampling routine of 40,000 parameter sets at intervals of 80 increments, and a burnin of 4,000. Demographic parameters were jointly estimated from the posterior sampling distributions: (1) Theta (*θ*), a measure of diversity proportional to the effective population size (*N_e_*) and mutation rate (*μ*) such that (*θ* = 4*N_e_μ*) and (2) the exponential growth rate parameter (*g*, with units of 1/generations) relative to the neutral mutation rate representing the direction and magnitude of change in population size. A Metropolis-coupled MCMC approach was employed for each run (using one cold chain and four heated chains). Acceptance rates fell between 5 and 40%, probability density functions were inspected for unimodality, and effective sample size (ESS) were confirmed to be >200 using Tracer v1.5 [91].

Past population dynamics were inferred using intron data with the Bayesian Skyline Plot (BSP) model in BEAST v1.6 [92]. A posterior distribution of effective population size through time was generated using a MCMC sampling scheme. Two independent analyses were run for 100,000,000 generations (sampling every 10,000 and 10% burnin) under a HKY substitution model, assuming a strict molecular clock with an approximated evolutionary rate of 0.002 substitutions/site/my for *β-Fibr* [93], [94]. We applied the piece-wise linear change model with 10 internodes. Skyline reconstruction was performed in Tracer v1.5 [91], and the median and 95% credibility interval were plotted as a function of time.

### Departures from neutrality and selection analyses

Hypothesis testing was conducted to check for significant departures from neutrality at ELA loci using approaches that reflect both recent and historical processes. All tests were conducted in Arlequin 3.5 [73] and significance determined with 10,000 simulations. First, we used Slatkin's Markov-Chain Monte Carlo (MCMC) implementation [95] of the Ewens-Watterson (E-W) Test [96], [97] to test for recent selection or demographic events (i.e. rapid population expansion or bottlenecks) affecting allele frequency patterns. The E-W test compared the observed homozygosity (*F*_obs_) with the expected homozygosity (*F*_exp_), based on a random sample of the same size consisting of the same number of alleles, simulated under the assumption of neutrality. Tajima's *D* [98] and Fu's *F*_S_ [99] were calculated for each population to test for departures from the null hypothesis of neutral evolution and population equilibrium. *D* and *F*_S_ are analogous to the E-W test, but because they are based on sequence data can also reflect historical selective pressures or demographic changes. *F*_S_ measures the probability of observing a certain number of alleles given the average pair-wise sequence divergence (*θ*), whereas Tajima's statistic contrasts the observed *θ* to that which would be expected under neutrality given the number of segregating sites (*S*). Negative values for both statistics imply population expansion or purifying selection, and positive values suggest either a population bottleneck or balancing selection. To tease apart signatures of demography and selection, we contrasted neutrality test results to those performed on *β-Fibr*. In addition, allele frequency distributions at neutral and functional loci were statistically compared across populations with contingency analyses in JMP 4.0 (SAS Institute Inc.).

To characterize molecular-level evidence of selection, we estimated rates of non-synonymous to synonymous mutations (*ω* = *d*_N_ */d*_S_) at coding genes (*DRA*, *DQA*) using maximum likelihood models of codon-substitution in the CODEML subroutine of PAML [100]. Input starting trees were generated using PhyML 3.0 [101] assuming the K80 +Γ_6_ (Kimura 2 parameter with 6 gamma-distributed rate categories) nucleotide substitution model as determined by jModelTest [102] and applying the subtree pruning and re-grafting (SPR) tree searching algorithm. We performed maximum likelihood ratio tests (LRTs) to test for significant positive selection by comparing the likelihoods of models of neutral evolution (M1a, M7) to those incorporating positive selection (M2a, M8). We also compared models assuming one evolutionary rate across codon sites (M0) to those allowing for heterogeneous rates (M3).

### Phylogenetic allele networks

We inferred phylogenetic relationships among sequence haplotypes by constructing a median-joining haplotype network [103] using maximum parsimony in Network 4.6.1 [104]. Allele frequency information and population proportion were incorporated into the visualization of the network. Unequal sample sizes were adjusted through rarefaction [105]. Sequences from the horse (*E. callabus*) were incorporated [GenBank: AY726647, L47174, M60100, L47172, AJ575295, FJ716134, L33909, U92505, U92506, U92507, U92508] to evaluate the ancestral state of *E. quagga* haplotypes.

## Results

### Microsatellite loci indices

Microsatellite loci evaluated in *E. quagga* were highly polymorphic, with a mean of 9.0 alleles per locus (range: 3–15) in the Etosha zebra population and 7.1 alleles per locus (range: 3–12) in Kruger (Table S3). Mean expected heterozygosity was 0.76 and 0.73 over all samples genotyped in Etosha and Kruger, respectively.

We found evidence for significant LD (*p*<0.001) between Hmb1 and Htg14 in Etosha, but not in Kruger. In addition, results from exact tests by locus and population indicated significant (*p*<0.001) heterozygote deficiency at Hms7 and Htg15, in Etosha only. We note that these observations may be due to the presence of null alleles. However, mean null allele frequency (NAF) by locus was estimated to be low (2.3%, range: 0–6.8%; Table S4). Total genotyping error varied by locus (range: 0–7%) and mean error rate was estimated to be 0.3% and 2.5% in blood and fecal samples genotyped, respectively. Breakdown of error contributions suggested allelic dropout only accounted for ∼0.5% of the error in fecal samples genotyped. Additional PCRs, following the comparative approach of Frantz *et al.* [59] lowered the error rate to 0%. Therefore, we are confident our genotyping approach yielded accurate results and that the assumptions made in subsequent analyses were upheld.

### Population genetic variation

Ten novel alleles were discovered at the *β-Fibr* locus in *E. quagga* [GenBank: KC109106–KC109115], with 8 polymorphic sites and two indel mutations totaling 14 base pairs. All *β-Fibr* alleles differed from a sequence previously reported in *E. callabus* [GenBank: AY726647]; However, β-Fibr*01 only differed by one nucleotide from the *E. callabus* sequence. Nine *DRA* alleles (Eqbu-DRA*01, *03-*05, *07-*11) [GenBank: AJ575299, EU930126, EU930121, EU930118, HQ637392–HQ637396] were recovered in Etosha and Kruger zebra populations. Twenty-one *DQA* alleles (Eqbu-DQA*01-02, *04-*22) [GenBank: EU935837, EU935829, EU935834–EU935836, EU935832, EU930130, HQ637397–HQ637409, KC109105] were also found. Population patterns of allelic richness were analogous with and without the correction applied for unequal sample sizes.

At the *DQA* locus, there was on average 1.56 (range: 1.31–1.80) and 1.77 (range: 1.46–2.07) alleles per individual in the Etosha and Kruger zebra populations, respectively. Less that 15% of samples cloned were found to have more than two alleles, and therefore the majority of our *DQA* data is likely derived from one locus. There was no evidence for significant differences in copy number frequency distributions across populations (*χ^2^* = 5.578, *p* = 0.1341; Figure S2).

**Figure 1:**
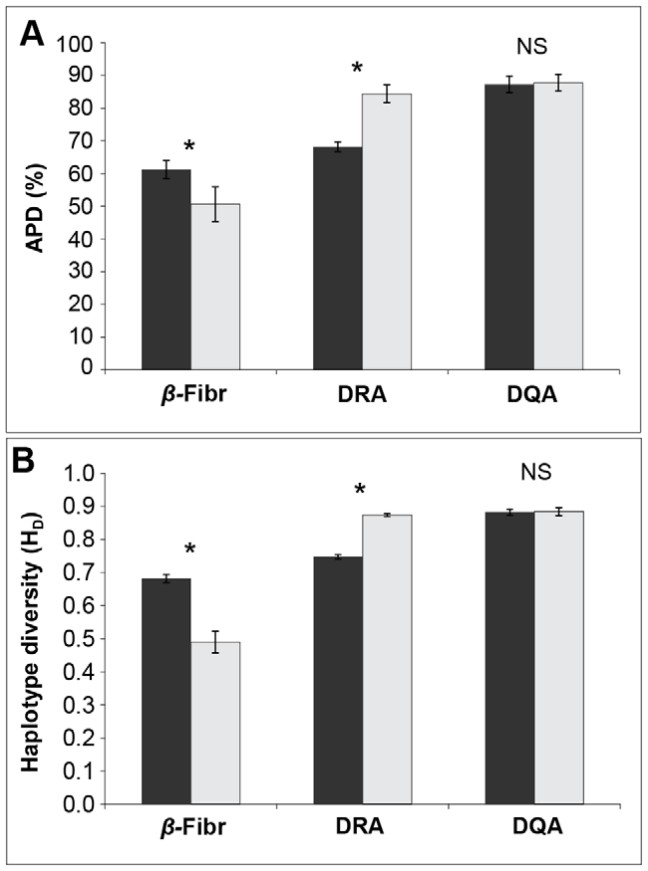
Statistical comparisons of diversity indices at *β-Fibr* and ELA loci (*DRA* and *DQA*) across populations. Genetic diversity indices plotted by population (Etosha: black, Kruger: gray) and locus. Diversity indices shown are: (A) average percent difference (APD), and (B) haplotype diversity (*H*_D_). Significant difference (*p*<0.05) between population means are indicated by an asterisk (*) and non-significance by NS.

At ELA loci, indices of sequence diversity (*H*_D_, *π* and *k*) were high when contrasted to *β-Fibr* intron data (Figure 1, Table 2). Comparisons of indices across populations revealed significantly depressed sequence diversity at the *DRA* locus in Etosha relative to Kruger (Figure 1). This pattern was consistent across sequence-based (*H*_D_, *π* and *k*) and allele frequency-based (*H_E_*) indices (Etosha: *H*_D_ = 0.748, *π* = 0.0088, *k* = 2.158, *H*_E_ = 0.743; *Kruger: H*_D_ = 0.874, *π* = 0.0097, *k* = 2.393, *H*_E_ = 0.860) (Table 2). This is also contradictory to what we would expect given the diversity at intron and microsatellite data— neutral diversity estimates were significantly higher in Etosha than Kruger. Diversity estimates at the *DQA* locus were similar across populations. Furthermore, results from non-parametric statistical analyses of APD, an unbiased diversity measure, corresponded with this inter-population pattern of variability (Figure 1, Table 3); APD in Etosha was significantly higher at the *β-Fibr* and lower at the *DRA* locus, whereas we could not reject the null hypothesis of APD mean equality across populations at the *DQA* locus. Thus, despite the potential bias in standard *DQA* diversity measures, the correspondence of APD patterns across loci and populations with other diversity estimates indicates our results are robust.

**Table 2:** Standard intra-population genetic diversity indices at neutral and ELA loci.

Population	Locus	*N*	*A*	*A*_CORR_	*H*_E_	*S*	*Indels (bp)*	*k*	*H*_D_ (*SD*)	*π* (*SD*)
**Etosha**	μsats (*n* = 13)^1^	84	8.92 (±0.87)	7.99	0.767 (±0.038)	—	—	—	—	—
	*β-Fibr* intron^2^	36	8	7.75	0.725	6	14	0.961	0.682 (0.037)	0.0015 (0.0002)
	*DRA*	72	8	7.84	0.743	9	0	2.158	0.748 (0.028)	0.0088 (0.0005)
	*DQA*	33	15	13.97	0.869	80	1	19.622	0.883 (0.026)	0.0962 (0.0073)
	*DQA*_ALL_	36	16	15.71	0.873	80	1	18.687	0.885 (0.025)	0.0916 (0.0073)
**Kruger**	μsats (*n* = 13)^1^	38	7.08 (±0.65)	7.01	0.749 (±0.033)	—	—	—	—	—
	*β-Fibr* intron^2^	24	6	6	0.517	4	14	0.553	0.490 (0.082)	0.0009 (0.0002)
	*DRA*	31	8	8	0.860	7	0	2.393	0.874 (0.013)	0.0097 (0.0005)
	*DQA*	23	13	13	0.865	78	0	14.739	0.884 (0.030)	0.0719 (0.0124)
	*DQA*_ALL_	30	15	15	0.891	81	0	17.165	0.897 (0.021)	0.0837 (0.0101)

Genetic diversity estimates are reported for microsatellites (μsats), the *β-Fibr* intron, and ELA loci (*DRA*, *DQA*). Indices include allelic richness (*A*), rarefaction-corrected allelic richness (*A*_CORR_), expected heterozygosity (*H*_E_), number of segregating sites (*S*), insertion/deletion mutations (indels) in base pairs (bp), average pair-wise difference between sequences (*k*), haplotype diversity (*H*_D_), and nucleotide diversity (*π*). The number of individuals (*N*) used in calculating diversity estimates are reported. Standard deviation (SD) or standard error (± SE) for estimates shown in parentheses. Diversity estimates for the *DQA* locus are reported both excluding (*DQA*) and including (*DQA_ALL_*) individuals with multi-locus genotypes.

1 *Mean (± SE) estimates reported, averaged over 13 loci.*

2 *A and H_E_ incorporate indel mutations in allele identification.*

**Table 3:** Unbiased intra-population genetic diversity indices independent of allele frequency.

	Etosha		Kruger		K-W test	Wilcoxon
Locus	*C*	APD ± SE	*C*	APD ± SE	*χ* ^2^, *p*-value	*Z*, *p*-value
*β-Fibr* intron	630	61.19±1.41	276	50.66±2.69	**18.45, ** ***p*** **<0.001**	**−4.295, ** ***p*** **<0.001**
*DRA*	2556	68.21±0.75	465	84.37±1.39	**75.80, ** ***p*** **<0.001**	**8.701, ** ***p*** **<0.0001**
*DQA*	528	87.25±1.29	253	87.75±1.85	0.28, *p* = 0.778	0.080, *p* = 0.778
*DQA_ALL_*	630	86.04±1.15	435	85.38±1.31	1.15, *p* = 0.284	−1.072, *p* = 0.284

Average percent difference (APD) and standard error (SE) are reported by population for *β-Fibr* intron and ELA loci. APD was estimated by pair-wise comparisons of all individuals in a population, with *C* equal to the number of comparisons made at each locus [72]. Chi-square (*χ^2^*) and *Z* statistics are reported for Kruskal-Wallis (K-W) rank randomization and Wilcoxon tests, respectively. Significant rejection (*p*<0.05) of the null hypothesis of group mean equality is indicated in boldface. Estimates for the *DQA* locus are reported both excluding (*DQA*) and including (*DQA_ALL_*) individuals with multi-locus genotypes.

### Population differentiation

Estimates of population pair-wise *F*_ST_ revealed evidence for significant, albeit low, levels of genetic differentiation at ELA loci across populations (*DRA*: *F*_ST_ = 0.045, *p*<0.001; *DQA*: *F*_ST_ = 0.02, *p* = 0.027; Table 4). *DQA* population structure was significantly lower than that observed at neutral loci, and the estimated *F*_ST_ fell outside of the microsatellite 95% CI (0.026–0.053). In contrast, *DRA* differentiation was not significantly different from *F*_ST_ estimates observed at microsatellite loci (*F*_ST_ = 0.038, *p*<0.001). Analyses of *β-Fibr* genetic structuring indicated substantially higher differentiation across populations (*F*_ST_ = 0.140, *p*<0.001) and supported the observation that low differentiation at ELA loci may be indicative of balancing selection acting on these genes. These results were corroborated by estimates of the *F*_ST_ analog, *F*′ (Table 4). The unbiased estimator of differentiation (*D*_est_) followed a similar pattern to that indicated by the patterns of *F*_ST_ across loci (Table 4) with of *β-Fibr* displaying higher levels of differentiation than both estimates based on microsatellites and ELA loci.

**Table 4:** Population differentiation estimates based on neutral and ELA loci.

Locus	*F*_ST_	*Φ*_ST_	Exact Test *p*-value	*F′* ± SE	*D*_est_
μsats (*n* = 13)^1^	0.038 (*p*<0.001)^2^	—	*p* = 1.000	—	0.101^3^
*β-Fibr* intron	0.140 (*p*<0.001)	0.129 (*p*<0.001)	*p*<0.001	0.178±0.027	0.282
*DRA*	0.045 (*p*<0.001)	0.051 (*p*<0.001)	*p*<0.001	0.034±0.013	0.193
*DQA*	0.020 (*p* = 0.027)	0.052 (*p* = 0.003)	*p*<0.001	0.013±0.017	0.153
*DQA_ALL_*	0.016 (*p* = 0.020)	0.018 (*p* = 0.046)	*p*<0.001	0.021±0.014	—

Conventional *F*-statistics (*F*_ST_), *Φ*_ST_ based on a K2P distance matrix, and global differentiation exact tests with *p*-values are reported. *F′*, an *F*_ST_ analog based on the similarity index [80], with standard error (± SE), and the Jost's *D* parameter (*D*_est_) are also shown. Divergence estimates for the *DQA* locus are reported both excluding (*DQA*) and including (*DQA_ALL_*) individuals with multi-locus genotypes.

1 *Estimates based on 13 loci.*

2 *95% confidence interval = 0.026–0.053.*

3 *Represents the harmonic mean of the estimated parameter over all loci.*

**Figure 2:**
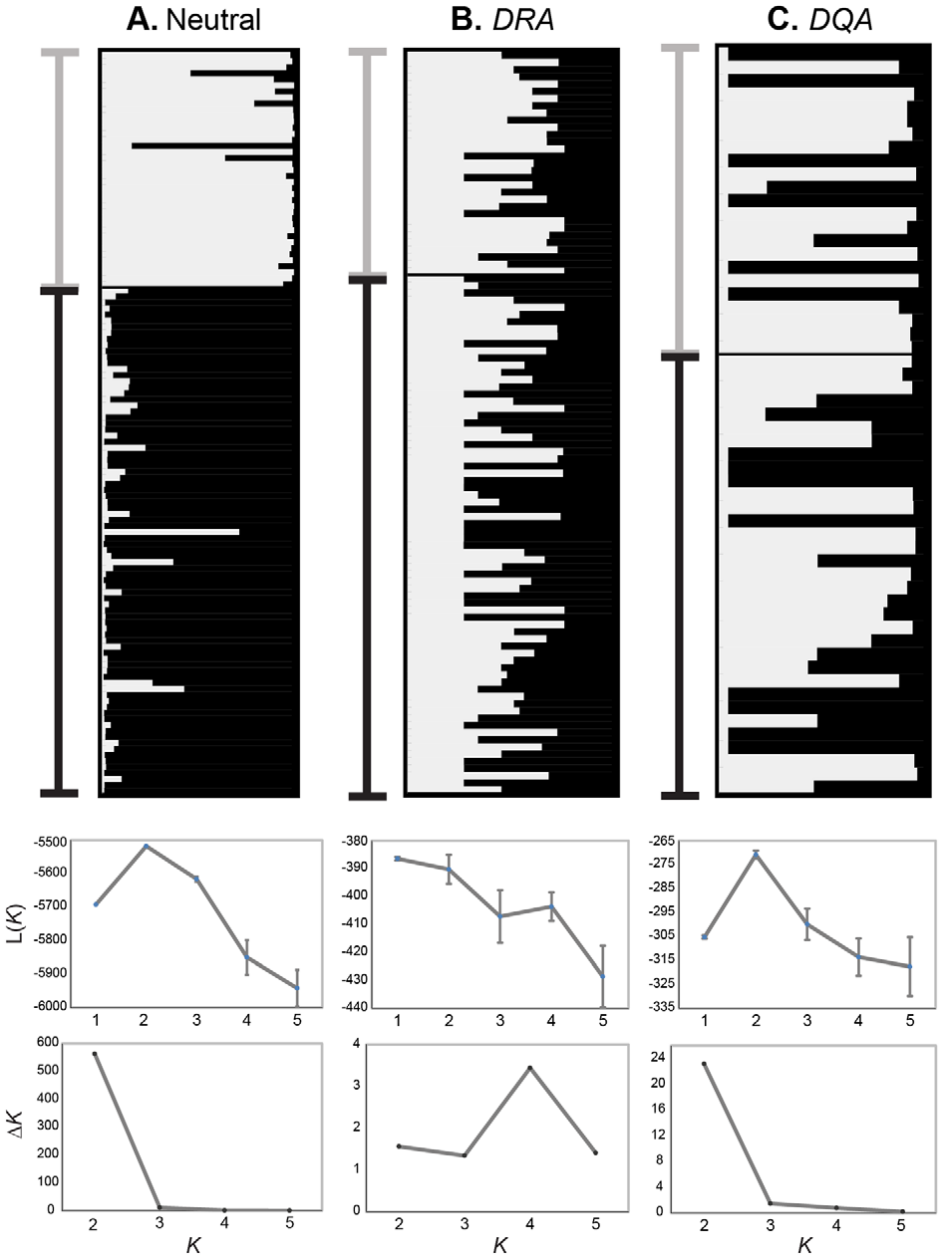
STRUCTURE plots of *E. quagga* from Etosha and Kruger. Individual genetic clustering assignments under the model of *K* = 2 based on (A) neutral loci (13 microsatellites and the *β-Fibr* intron), (B) ELA-*DRA*, and (C) ELA-*DQA*. Percent assignment to each of two genetic clusters are shown for each individual genotype in plots. Population where an individual was sampled is indicated by the bar to the left of each plot (Etosha: black, Kruger: gray). The mean posterior probability (L(*K*)) ± 95% confidence interval and the rate of change in the log probability of the data between successive *K* values (Δ*K*) from *K* = 1 to 5, for each marker type are shown below each corresponding STRUCTURE plot.

Clustering analyses in STRUCTURE based on neutral data revealed that a model of *K* = 2 populations had the highest likelihood (Ln = −5518.11) and highest Δ*K*, with individuals from Etosha clustering separately from those in Kruger (Figure 2, Table S5). At the *DQA* locus, the best-fit model was similarly *K* = 2 genetic clusters but, in contrast, clustering did not correspond to population. At the *DRA* locus, *K* = 1 had the highest log likelihood, but the highest Δ*K* suggested *K* = 4 populations. However, Δ*K* is not able to find the best *K* when the true *K* = 1 [83] and inspection of STRUCTURE plots under models of *K*≥2 showed that clustering assignments broke down within individuals.

### Demographic inference from neutral data

Mismatch distribution analyses of the neutral intron indicated a weak signature of population expansion in the Etosha zebra population, as the null model could not be rejected (SSD = 0.014, *p* = 0.058). The model of sudden population growth was rejected in Kruger, but with marginal significance (SSD = 0.029, *p* = 0.043). As significance is borderline for both populations, mismatch results are inconclusive. However, in both Etosha and Kruger the population expansion model could not be rejected as indicated by relatively low, non-significant estimates of the Harpending's raggedness index (Etosha: *RI* = 0.085, *p* = 0.11; Kruger: *RI* = 0.128 *p* = 0.39).

**Figure 3:**
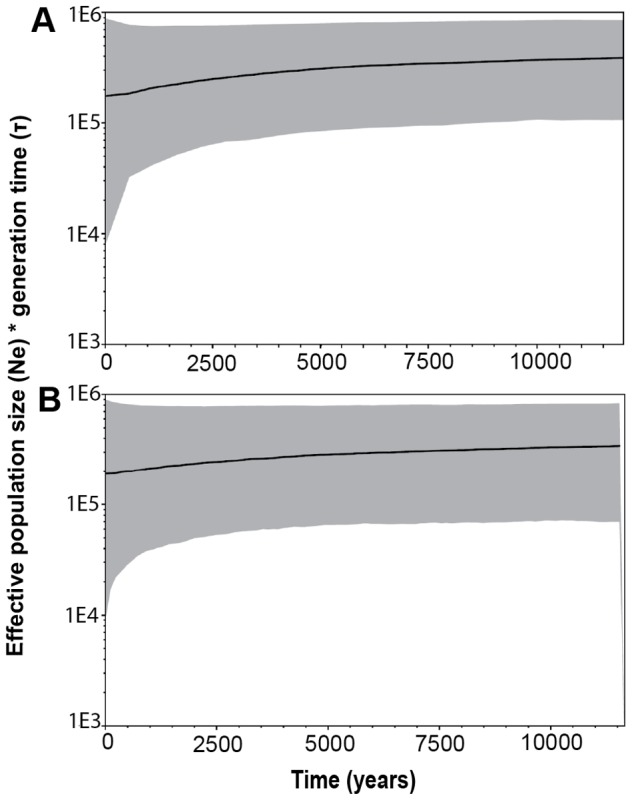
Bayesian skyline plots showing changes in population size over time. Median estimates of effective population size (*N_e_*), relative to generation time, is plotted over time (years before present) for (A) Etosha and (B) Kruger zebra populations. The 95% credibility interval is shown by gray shading.

Outcomes from demographic analyses in LAMARC similarly indicated low positive growth, of similar magnitude in both populations. The most probable estimate for the exponential growth rate parameter (95% confidence interval) in Etosha was 0.302 (0.253–0.549) generations^−1^, whereas, in Kruger, *g* was estimated to be 0.388 (0.317–0.667) generations^−1^. Although positive, the magnitude of *g* is extremely small and may indicate populations that are in the process of stabilization. The most probable estimate of the diversity measure, *θ*, was identical across populations, estimated to be 99.3 (84.8–100.2) and 99.3 (83.0–100.1) in Etosha and Kruger, respectively. Bayesian skyline plots, based on an independent dataset, confirmed that these zebra populations are both large, and have similarly experienced stability in the recent past (Figure 3).

### Selection analyses

Tajima's *D* and Fu's *F*_S_ tests for departures from neutrality were not significant (*p*>0.05) at all loci and populations (Table 5). Although non-significant, test statistics were negative at the intron, indicating a potential weak effect of population expansion. Positive values observed at ELA loci contrasted neutral data and may be representative of positive selection acting on the site frequency spectrum. Results from Slatkin's E-W test revealed significantly lower homozygosity (*F*_obs_) than would be expected under a neutral model, supporting the hypothesis that balancing selection is occurring at ELA loci. However, when multi-locus genotypes are excluded at the *DQA*, these results become non-significant. Significance of E-W tests, but not *D* or *F*_S_, implies that selection at these loci is possibly a relatively recent phenomenon.

**Table 5:** Tests of neutrality and population equilibrium.

Population	Locus	*N*	*D*	*F*_S_	E-W-S Test	
					*F*_obs_	*F*_exp_
**Etosha**	*β-Fibr* intron	72	−0.532	−1.681	0.269	0.312
	*DRA*	144	0.780	1.304	0.257*	0.361
	*DQA*	66	0.571	11.214	0.131	0.153
	*DQA_ALL_*	76	0.486	10.882	0.127*	0.149
**Kruger**	*β-Fibr* intron	47	−0.895	−1.776	0.325	0.371
	*DRA*	62	1.538	0.550	0.140*	0.299
	*DQA*	46	−0.604	6.324	0.135	0.160
	*DQA_ALL_*	67	0.040	9.380	0.117*	0.156

Neutrality at the *β-Fibr* intron and ELA loci was assessed by population based on Tajima's *D* (*D*), Fu's *F*_S_ (*F*_S_), and the Ewens-Watterson-Slatkin (E-W-S) test. The number of alleles (*N*) used for each locus and population are reported. Significant rejection of the null hypothesis of neutrality or constant population size is indicated in boldface (*p<0.05). Estimates for the *DQA* locus are reported both excluding (*DQA*) and including (*DQA_ALL_*) individuals with multi-locus genotypes.

**Figure 4:**
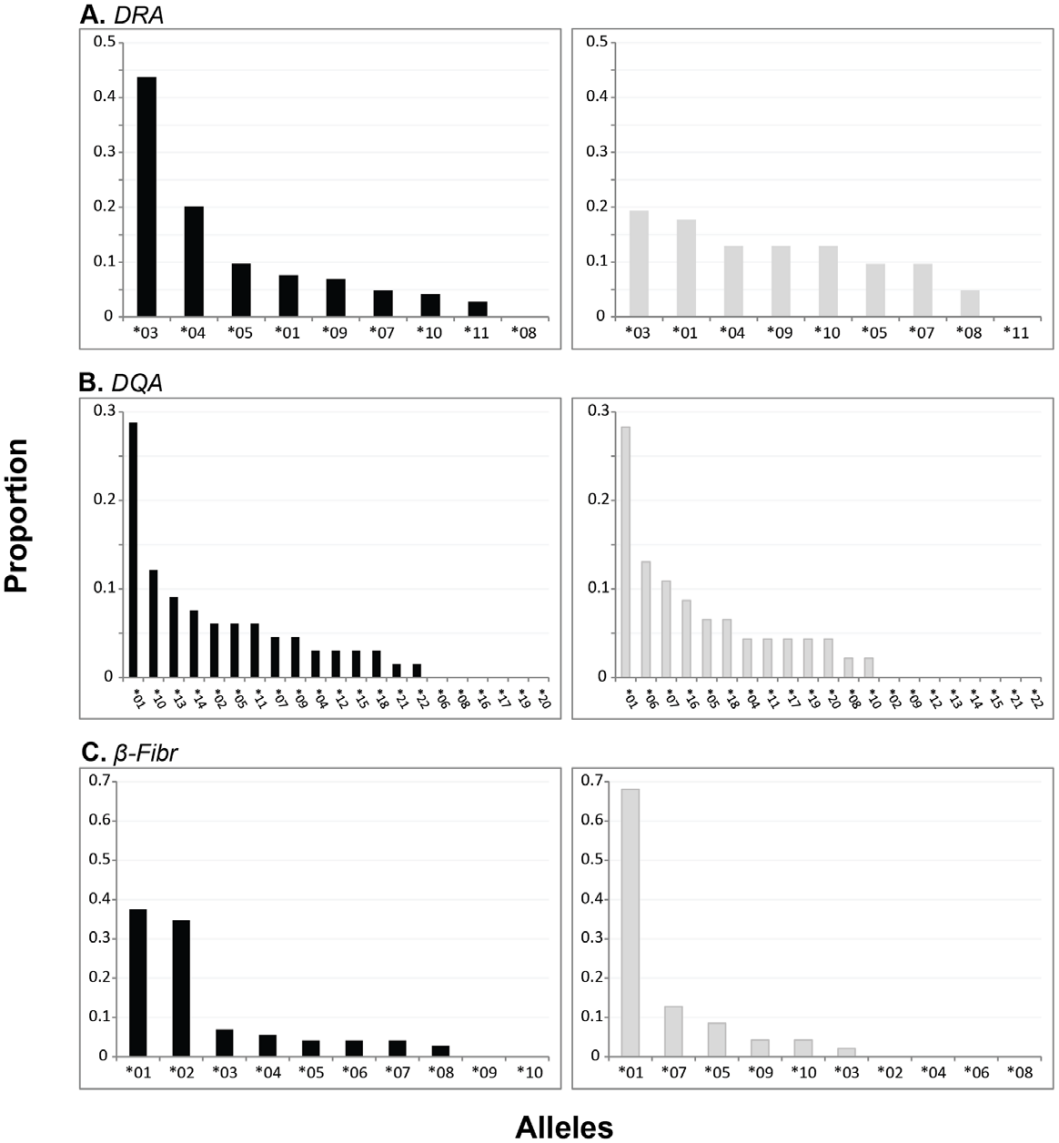
Allele frequency distributions by locus and population. Alleles presented in order of descending frequency at the (A) ELA-*DRA*, (B) ELA-*DQA*, and (C) *β-Fibr* intron by population, with Etosha shown in black and Kruger in gray. Allele sample sizes are as follows: Etosha (*β-Fibr*: 72, *DRA*: 144, *DQA*: 66) and Kruger (*β-Fibr*: 47, *DRA*: 62, *DQA*: 46). *DQA* allele frequencies were calculated excluding individuals with multi-locus genotypes.

Skewness in population allele frequency distributions may be interpreted as a signature of demographic change and/or selection, and was used to tease apart findings from neutrality tests. ELA allele frequency distributions were inspected relative to that at the *β-Fibr* by population. The most outstanding observation was that *DRA* allele frequencies were evenly distributed in Kruger, but skewed in Etosha (Figure 4), with contingency analyses indicating significant differences in frequency distributions across populations (*χ^2^* = 15.4, df = 7, *p* = 0.03). In particular, the DRA*03 allele was predominant in Etosha, representing approximately 44% of all alleles observed which could potentially be driving the decreased diversity found at this locus. Contingency analyses also suggested significantly different allele frequency distributions at the *DQA* locus (*χ^2^* = 62.1, df = 20, *p*<0.001). *DQA* distributions differed in the large number of rare and private alleles found in each population (Figure 4, Figure S3). In both populations, the DQA*01 allele was present at a significantly greater frequency than any other allele (25–29%). *β-Fibr* allele frequency distributions were also significantly different (*χ^2^* = 51.9, df = 9, *p*<0.001) between populations, with Kruger exhibiting a more skewed distribution (Figure 4).

Molecular-based analyses revealed no evidence for positive selection or variable rates of selection at the molecular level of the *DRA* locus in both zebra populations (Table 6). In contrast, at the *DQA* locus, likelihood ratio tests (LRTs) comparing models of invariable versus variable evolutionary rates across codon sites (M3 *vs.* M0) and models of positive selection to neutral evolution (M7 *vs.* M8) were significant (*p*<0.05) in both populations. While estimates of *ω* across the *DQA* gene region were close to 1 in both populations (Etosha: *ω* = 1.11, Kruger: *ω* = 0.71), approximately 40% of codon sites were determined to be under significant positive selection (*ω* = 2.52).

**Table 6:** Molecular detection of selection across codon sites at the ELA-*DRA* and *DQA*.

Locus	Model comparison	df	Etosha			Kruger		
			*ω*	*χ^2^*	*p- value*	*ω*	*χ^2^*	*p- value*
***DRA***	M0 v. M3	4	0.213	6.60E-04	1.000	0.249	3.80E-04	1.000
	M1a v. M2a	2		2.40E-05	1.000		1.00E-04	1.000
	M7 v. M8	2		−4.18	1.000		4.00E-04	1.000
***DQA***	M0 v. M3	4	1.112	**50.10**	**0.000**	0.707	**64.23**	**0.000**
	M1a v. M2a	2		**13.36**	**0.001**		**10.99**	**0.004**
	M7 v. M8	2		**13.76**	**0.001**		**11.98**	**0.002**
***DQA_ALL_***	M0 v. M3	4	1.082	**51.68**	**0.000**	0.681	**59.14**	**0.000**
	M1a v. M2a	2		**13.16**	**0.001**		4.93	0.085
	M7 v. M8	2		**14.56**	**0.001**		**7.54**	**0.023**

Model comparisons and likelihood ratio tests (LRTs) were conducted to test for significant heterogeneity across sites (M3 v. M0) and positive selection (M1a v. M2a, M7 v. M8). Significance (*p*-value) of LRTs was determined by calculation of the chi-square test statistic (χ^2^) and degrees of freedom (df). Significant test results (*p*<0.05) are highlighted in boldface. Non-synonymous to synonymous substitution rates (*ω*) at each locus and population were estimated from M8 in PAML [100]. Estimates for the *DQA* locus are reported both excluding (*DQA*) and including (*DQA_ALL_*) individuals with multi-locus genotypes.

### Phylogenetic relationships among alleles

**Figure 5:**
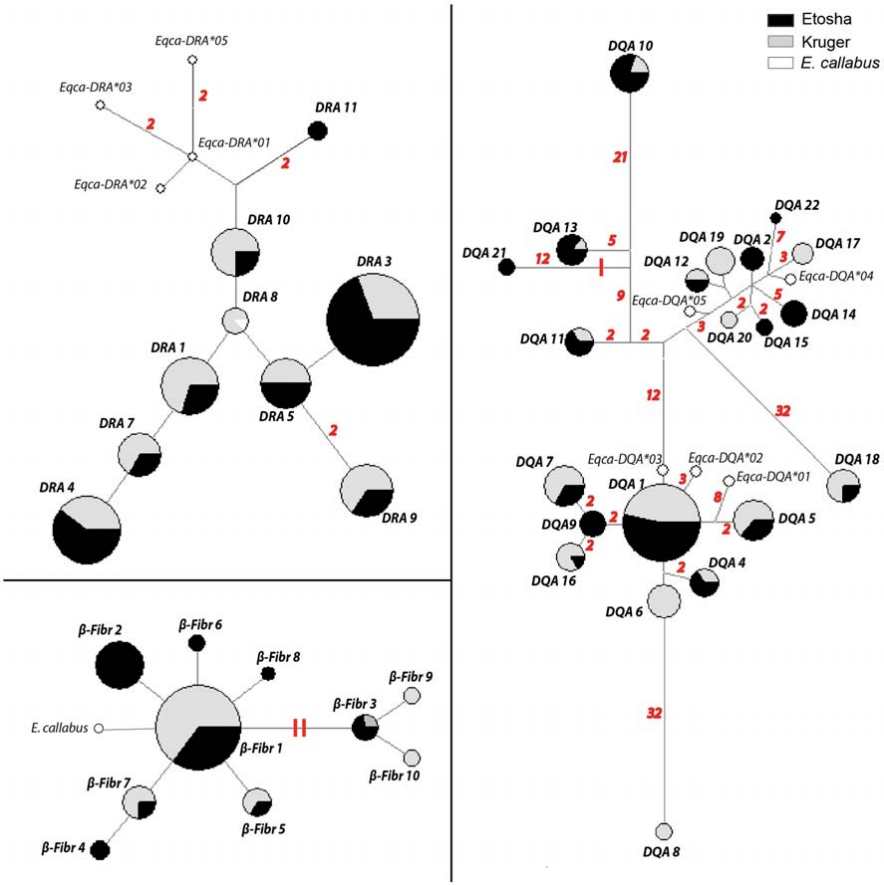
Median-joining haplotype networks for ELA*-DRA*, ELA*-DQA*, and *β-Fibr* loci. Circle size is proportional to haplotype frequency. Proportion of total alleles found in Etosha (black) and Kruger (gray) are shown, with sample sizes corrected by rarefaction. Haplotypes from *Equus callabus* are shown in white (GenBank accession numbers– *β-Fibr:* AY726647, *DRA:* L47174, M60100, L47172, AJ575295, FJ716134, *DQA:* L33909, U92505, U92506, U92507, U92508). Differences between haplotypes that were greater than one mutational step are notated in red italics. Insertion-deletion mutational events are represented by a red perpendicular line.

Phylogenetic median-joining haplotype networks of ELA loci showed a lack of geographical allele structuring by population (Figure 5). The *β-Fibr* allele from *E. callabus* differed by one mutational step from the most frequent allele observed in *E. quagga* (β-Fibr*01). At the *DRA*, four *E. callabus* alleles were ancestral to the Eqbu-DRA*10 and Eqbu-DRA*11 alleles in *E. quagga*. However, one allele was shared between the two species (Eqbu-DRA*08 = Eqca-DRA*04). At the *DQA*, there were large mutational differences observed between alleles, and *E. callabus* alleles were found distributed throughout the network.

## Discussion

This study juxtaposed MHC with neutral data to disentangle the co-occurring effects of selection and neutral evolutionary processes (e.g. mutation, gene flow, changes in population size, and drift) on the distribution of immunogenetic variation across *E. quagga* populations. Our results suggested that selection is acting variably across MHC genes and populations in zebra.

Demography and migration dynamics may confound our ability to detect selection. We used neutral data to elucidate population history and extricate the role of these processes in shaping immune gene variation. Population size estimates from plains zebra in Kruger (1980–1993) [47], [48] and Etosha (2000–2012) [Namibian Ministry of Environment and Tourism, unpublished data] suggest that zebra populations in both regions have remained relatively stable. This is in agreement with Bayesian skyline plots and demographic estimates (*g*) from this study that indicated effective population trajectories are similar in Etosha and Kruger zebra and suggested historically stabilized populations with the possibility of low, positive growth. Given census estimates, we expected neutral genetic diversity to be higher in Kruger due to a population size that is approximately twice as large as Etosha. In contrast, we found the opposing pattern, with neutral diversity being higher in Etosha. This discrepancy could be due to sampling artifacts from differences in survey approaches and observer bias across parks, or it is possible there is subdivision within the Kruger zebra population and our genetic data represent only a portion of this population. To date, however, there has been no genetic study in zebra documenting the degree of population mixing throughout Kruger.

At microsatellite loci, genetic differentiation across zebra populations was relatively low (*F*_ST_ = 0.04). This finding is in agreement with a recent phylogeographic study on five subspecies of *E. quagga* that showed zebra population structuring is among the lowest of 17 different savannah-adapted ungulate species in Africa (mtDNA: *Φ*_ST_ = 0. 17, microsatellites: *θ*_ST_ = 0.05) [106]. Population structure at the *β-Fibr* intron (*F*_ST_ = 0. 14, *Φ*_ST_ = 0. 13), however, was higher than that based on microsatellites (*F*_ST_ = 0.04). This discrepancy between nuclear markers suggests further inquiry into the possibility that our assumption of the neutrality at the *β-Fibr* intron has been violated. Alternatively, the differences observed between structure as inferred from microsatellite and intron loci may be due to differences in the mutational modes and rates of these markers [107].

Under balancing selection, due to similar pathogen regimes across populations, we would expect low population differentiation at MHC loci relative to neutral loci due to theoretically greater effective migration rates [108]. This has been shown to occur even among small populations with little gene flow that are highly susceptible to drift effects [12]. In contrast to this, if the mode of selection varies among populations, we may expect MHC divergence to be higher than that estimated from neutral markers. Consistent with the prediction of a gene under balancing selection, we found that *F*_ST_ measures at the *DQA* locus were seven to eight times lower than at the *β-Fibr* intron, and two to three times lower than microsatellite loci. At the *DRA* locus, *F*_ST_ was two times lower than that estimated at the intron. It is possible these results are driven by high heterozygosity at this locus, given that *F*_ST_ estimates may decrease as locus variability increases [77], [78]. Unbiased estimates of differentiation (Jost's *D*) mirrored *F*_ST_ results in comparisons of ELA loci with the *β-Fibr* intron, suggesting that the pattern may also be upheld. Furthermore, STRUCTURE results using neutral data revealed clustering of individuals by population, in contrast to the lack of geographic clustering observed at both ELA loci.

Differentiation at the *DRA* locus was comparable to estimates at microsatellite loci. A similar observation was made at the *DRA* locus among domestic donkey (*E. asinus*) populations [109]. Also, previous studies have contradicted expectations with discoveries of comparable or elevated population structure at MHC versus microsatellite loci [9], [10], [14], [32]. This observation may be attributed to either (*i*) local adaptation, (*ii*) weak selection acting on the MHC locus, or (*iii*) an artifact of using genetic markers evolving under different mutational modes and rates [107]. Our finding could be due to one or a combination of the aforementioned causes. Reduced structure at the *DRA* relative to the *β-Fibr* locus, akin to our observations at the *DQA* locus, indicates that a difference in the mutation model may be responsible for the discrepancy. But, the lack of a similar observation at the *DQA* may alternatively suggest that local adaptation is occurring at this locus. We note, however, that our inferences regarding demography based on a single locus must also be taken with caution.

Despite being low, ELA population differentiation remained significant, as indicated by pair-wise *F*_ST_ and global exact tests. The evolutionary process of genetic drift may be affecting ELA differentiation, albeit weakly, by stochastically shifting allele frequency distributions within populations. This is parsimonious with an expectation of little to no recent gene flow between these two populations, as they are contained in parks and separated by more than 2,000 km of a human-dominated matrix. An alternative explanation (to that of genetic drift) is that variable pathogen pressures across the localities and over time may be driving distributional shifts in ELA allele frequencies across populations.

The *E. quagga* population in Etosha exhibited higher genetic diversity than Kruger at all neutral loci. This pattern was fairly consistent, regardless of the diversity index evaluated. In contrast, *DQA* population diversity patterns are generally similar across populations. It is possible that balancing selection may be preserving similar levels of *DQA* diversity due to equivalently high pathogen diversity across populations. Interestingly, *DRA* genetic diversity across populations is strikingly incongruent with that observed at neutral loci, revealing depressed variability in Etosha. Allele frequency distributions showed that the paucity of diversity at this locus in Etosha is driven by the presence of one predominant allele. In contrast, Kruger zebra possessed many of the same alleles, but frequencies were relatively evenly distributed. Opposing *DRA* and neutral diversity patterns across populations provides evidence for geographically heterogeneous selection at the *DRA*. Pathogen-mediated selective pressure may differentially affect these two loci because of differences in their functional role (i.e. differences in the specific pathogens that these antigens are capable of recognizing).

Specifically, the marked difference in *DRA* patterns across populations, with respect to neutral data, highlights the possibility that zebra in Etosha may be subject to local selective pressure by pathogens at this locus. Matthee *et al.* [41] found that gastrointestinal helminth communities differed significantly among African equids and even between Etosha and Kruger zebra populations, signifying that the specialization of parasite communities has followed host population divergence. Notably, Etosha zebra had a considerably lower Stronglyinae nematode species richness— with only one species as opposed to six identified in Kruger [41]. Despite low species diversity in Etosha, nematode prevalence has been found to be extremely high (>98%: [40]). Etosha's particularly arid climate (rainfall<500 mm/year) relative to Kruger's (rainfall 550–650 mm/year) may play an important role in limiting macroparasite diversity [41].

Besides intestinal parasites, zebra in Etosha are also known to be severely affected by anthrax, a lethal infectious bacterial disease [36], [37]. Zebra in the park exhibit the highest recorded incidence of anthrax in southern Africa and the disease has been implicated as one of the primary causes of adult mortality [36]. In contrast, Kruger zebra experience infrequent and less severe anthrax outbreaks [38]. Given the role of MHC class II genes in recognizing extracellular pathogens, we speculate that the combined low, but prevalent parasite community and repeated severe anthrax outbreaks may present significant selective pressures limiting MHC diversity in Etosha.

Tajima's *D* and Fu's *F*_S_ tests were unable to reject the hypothesis of neutral evolution; However, we recognize that the ability of these tests to detect selection depends on the nature of the mutational process and the duration, strength and timing of selection [110]. Demographic processes may obscure signatures of selection and it is unclear whether this signature has been preserved in the current variation in these genes. Despite lack of significance, the sign of *D* and *F*_S_ test statistics were generally consistent with positive selection at ELA genes and opposed values observed at the intron, indicating a putative weak selection signal. As these statistics reflect selection operating to change the site frequency spectrum, accumulation of such mutations may require long time periods extending beyond the history of a population. Whereas, the significant E-W tests recovered here support the conclusion that relatively recent balancing selection plays an important role in shaping allele frequency distributions within zebra populations. At the *DQA* locus, molecular selection analyses also suggested significant heterogeneity across the gene region and positive selection at approximately 40% of codon sites. Thus, while balancing selection is likely maintaining diversity at these genes, it is also possible that selection signatures have been obscured over time by fluctuating selective pressures due to changing pathogen communities or demographic events.

Simulations by Ejsmond *et al.* [111] demonstrated that allele frequency distributions are not always predictable under balancing selection, depending on the specific mode in effect (e.g. overdominance, negative frequency-dependence). Under overdominant selection, allele frequency distributions are consistently more even than those observed under neutrality. Whereas, with negative frequency-dependence, distributions are unpredictable and can be skewed, even, or indistinguishable from that observed under neutrality, depending upon at what point during the host-parasite cycle sample collection occurred. The conclusion is that it is impossible to indisputably infer mode of selection from neutrality tests based on departures of allele frequency distributions from neutral expectations (e.g. the E-W test). Therefore, we cautiously conclude balancing selection is occurring at these loci based on results from E-W tests, and do not make any further interpretation regarding the strength or nature of selection.

The hypothesis that balancing selection preserves MHC diversity is well accepted and supported in the literature [20], [21]. Furthermore, recent studies focused on long-term evolutionary patterns (through evaluation of *d*_N_/*d*_S_ ratios and MHC gene phylogenies) have provided genus-level evidence for balancing selection acting on both the *DRA* and *DQA* loci in equids [44], [45]. Our results provided several lines of evidence to further support its occurrence over shorter evolutionary time scales in zebra (i.e. over the history of the species or population), including (*i*) elevated ELA diversity over neutral diversity within populations, (*ii*) low genetic structure across populations relative to that observed at neutral loci, (*iii*) significant rejection of the null hypothesis of neutrality based on the assumption of mutation-drift equilibrium, and (*iv*) evidence for heterogeneous rates of evolution across codon sites, with significant positive selection occurring at specific sites (only at the *DQA*). These combined findings provide convincing evidence for balancing selection operating on ELA genes in zebra. However, the somewhat differing patterns observed at the *DRA* across populations also suggest that there may be heterogeneous selection pressures and local adaptation operating at this locus.

## Conclusions

MHC studies in natural populations are critical for understanding adaptation and evolutionary potential, as variation in these genes reflect biologically relevant processes significant to fitness. Patterns of MHC variation are shaped by a complex interplay of selective and demographic factors, which may be challenging to disentangle, but possible to achieve through the amalgamation of multiple lines of evidence. Our data suggest that selection on MHC genes may vary spatially, and also differ by locus. Balancing selection over evolutionary time scales may act cumulatively to retain MHC diversity, but this selection signature may be obscured due to fluctuating and diverse pathogen communities. We found evidence for balancing selection at MHC genes in zebra populations, but we also conclude heterogeneous selection may be acting across populations at the *DRA* locus— two findings which are compatible when considering different time and spatial scales. These results highlight the importance of integrating neutral and adaptive data over different scales to uncover the relative effects of demography and selection in shaping functional diversity. Future ecological studies are warranted that investigate the link between host immunogenetic diversity and pathogen community structure to better understand the mechanisms underlying adaptation.

## Supporting Information

Protocol S1 **Polymerase Chain Reaction protocols for neutral loci.** PCR reagents and cycling conditions used to amplify the *β-Fibrinogen*, *intron 7* and microsatellite loci: Aht21, Asb23, Cor014, Hmb1, Hms7, Htg7, Htg9, Htg14, Htg15, Lex20, Lex33, Lex52, Ucdeq505, Um011, and Vhl47.
(DOC)

Figure S1 **Flowchart of comparative microsatellite genotyping approach.** This approach involves comparing two to three initial replicate PCRs, with heterozygotes confirmed in two PCRs and homozygotes in three PCRs. If a disagreement is found (e.g. first PCR results in heterozygote, and second in homozygote for one allele) additional PCRs were performed until each allele is observed a minimum of two times. In the event that no consensus was found, an individual was either scored as having a missing genotype or given a half-locus genotype, by assigning one allele as missing data. In summary, a minimum of 2 PCRs is required to confirm a heterozygote genotype and 3 PCRs for a homozygote, with a maximum of up to 7 PCRs conducted. Adapted from Hansen *et al.* [61].
(TIF)

Figure S2 **Frequency distribution of ELA-** ***DQA*** **copy number.** *DQA* copy number frequency distribution in individuals from Etosha (black) versus Kruger (gray). One to four alleles were observed in each individual. Contingency goodness-of-fit analyses revealed no significant difference between population frequency distributions (*χ^2^* = 5.578, *p* = 0.1341).
(TIF)

Figure S3 **ELA-** ***DQA*** **allele frequency distributions, including alleles from multi-locus genotypes.** Alleles are presented in descending order and by population, with Etosha in black and Kruger in gray.
(TIF)

Table S1 **Populations sampled and individuals successfully genotyped at each locus.** Loci investigated include microsatellites (μsats), *β-Fibrinogen* intron 7 (*β-Fibr*), ELA-*DRA* exon 2 (*DRA*), and ELA-*DQA* exon 2 (*DQA*). The number of confirmed alleles found in each population is reported, with that for μsats reported as the mean (standard error) of 15 loci. Over both populations, typing success ranged from 41–71% per locus.
(DOC)

Table S2 **Description of microsatellite loci.** Includes total number of alleles, observed size range in base pairs (bp), fluorescent label used for genotyping analyses, annealing temperature used in polymerase chain reaction (PCR), and the reference study in which it was initially discovered in *E. callabus*.
(DOC)

Table S3 **Microsatellite diversity by locus and population.** For each population, diversity by locus and total mean diversity are reported. Diversity is described in terms of number of alleles (*A*), observed heterozygosity (*H*_O_), and expected heterozygosity (*H*_E_). Sample sizes (*N*) are reported for each locus and population. Significant departures from Hardy-Weinberg equilibrium are indicated in boldface.
(DOC)

Table S4 **Microsatellite null allele frequency and genotyping error rates.** Null allele frequency (NAF) was estimated by population and locus. Total genotyping error rates, broken down by allelic dropout and false allele generation, were determined by paired genotyping of blood and fecal samples from individual zebra captures. All rates marked as “0” represent values of 0.000.
(DOC)

Table S5 **Results from clustering analyses in STRUCTURE.** Analyses were conducted over 10 runs of *K* = 1 to 5, combining all neutral data (13 microsatellites and the *β-Fibr* intron) and, separately, at each ELA locus. The mean posterior log probability (Mean *L*(*K*)), standard deviation (SD *L*(*K*)), mean difference between successive likelihood values of *K* (*L′*(*K*)), absolute value of the difference between successive values of *L′*(*K*) (|*L″*(*K*)|), and second order rate of change of the likelihood function with respect to *K* (Δ*K*) are reported. The best-fit models are highlighted in bold, and were selected based on mean posterior probabilities and following the approach described by Evanno *et al.* [83].
(DOC)
