# Supplementary material for: Unraveling the Effects of Selection and Demography on Immune Gene Variation in Free-Ranging Plains Zebra (Equus quagga) Populations
Source: PLoS One. 2012 Dec 14;7(12):e50971. doi: 10.1371/journal.pone.0050971 (PMC3522668; doi:10.1371/journal.pone.0050971)
Supplement: Protocol S1 — Polymerase Chain Reaction protocols for neutral loci. PCR reagents and cycling conditions used to amplify the β-Fibrinogen, intron 7 and microsatellite loci: Aht21, Asb23, Cor014, Hmb1, Hms7, Htg7, Htg9, Htg14, Htg15, Lex20, Lex33, Lex52, Ucdeq505, Um011, and Vhl47. (DOC) [file pone.0050971.s001.doc]

**Protocol S1. Polymerase Chain Reaction protocols for neutral loci**

*β-Fibrinogen, intron 7*

PCR mixes contained 1.75uL of 10x PCR buffer (100mM Tris-Cl, pH 8.3, 500mM KCl, 15mM MgCl_2_, 0.01% (w/v) gelatin) 0.4 mM deoxynucleotide triphosphates (dNTPs), 1U Ampli*Taq* Gold DNA polymerase (Applied Biosystems), 15µg bovine serum albumin (BSA) (New England BioLabs), 0.67 µM of each primer, and 25-50ng of DNA in a 15µL total reaction volume. The following thermocycling conditions were used: an initial denaturation at 95ºC for 6 min; 48 cycles of 94ºC for 1 min, 62ºC for 1 min 30 s, and 72ºC for 2 min; final extension at 72ºC for 10 min.

*Microsatellites*

Each locus was amplified by PCR in a 10 µL total reaction volume, comprising of approximately 25ng DNA, 1.33 µL GeneAmp 10x PCR buffer, 0.7 U Ampli*Taq* Gold DNA polymerase (Applied Biosystems), 0.4 mM dNTPs, 10µg BSA (New England BioLabs) and 0.50 µM of each primer. PCRs for Aht21, Cor014, Hmb1, Hms7, Htg15, and Lex52 were carried out under a “touch-down” thermocycling profile: initial denaturation at 95ºC for 10 min; 2 cycles of 94ºC for 1 min, 60ºC for 1 min, and 70ºC for 35 s; 18 cycles of 93ºC for 45 s, 59ºC for 45 s, and 70ºC for 45 s, with the annealing temperature decreasing by 0.5ºC with each cycle; 30 cycles of 92ºC for 30 s, 50ºC for 30 s, and 70ºC for 1 min; final extension at 72ºC for 10 min. Whereas, PCRs for the remaining microsatellite loci used the following profile: initial denaturation at 95ºC for 6 min; 40 cycles of 94ºC for 1 min, 55-60ºC for 1 min, and 72ºC for 45 s; final extension at 72ºC for 5 min (Table S2).
