# Supplementary figures and images for: Unraveling the Effects of Selection and Demography on Immune Gene Variation in Free-Ranging Plains Zebra (Equus quagga) Populations
Source: PLoS One. 2012 Dec 14;7(12):e50971. doi: 10.1371/journal.pone.0050971 (PMC3522668; doi:10.1371/journal.pone.0050971)

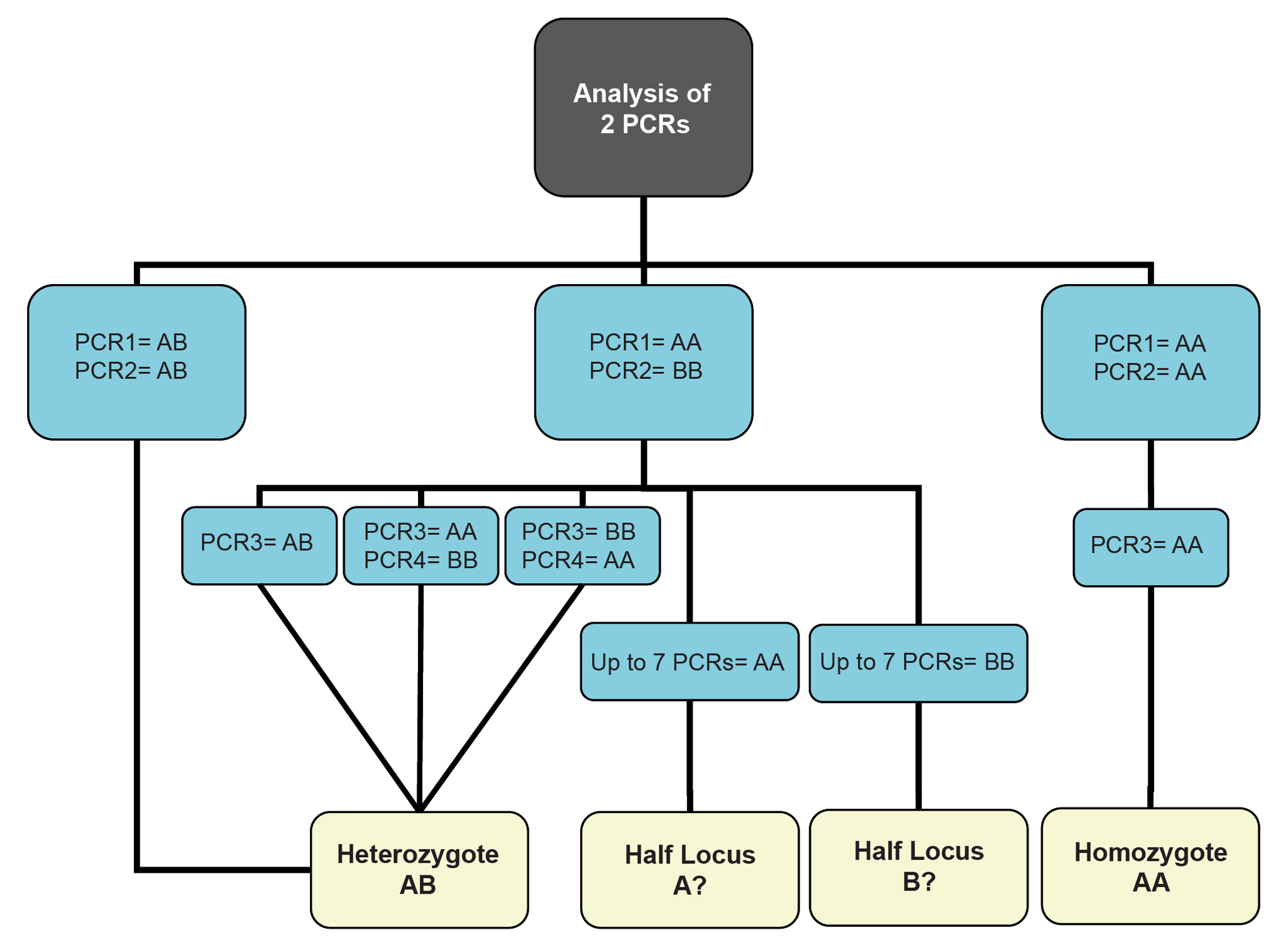

Supplement: Figure S1 — Flowchart of comparative microsatellite genotyping approach. This approach involves comparing two to three initial replicate PCRs, with heterozygotes confirmed in two PCRs and homozygotes in three PCRs. If a disagreement is found (e.g. first PCR results in heterozygote, and second in homozygote for one allele) additional PCRs were performed until each allele is observed a minimum of two times. In the event that no consensus was found, an individual was either scored as having a missing genotype or given a half-locus genotype, by assigning one allele as missing data. In summary, a minimum of 2 PCRs is required to confirm a heterozygote genotype and 3 PCRs for a homozygote, with a maximum of up to 7 PCRs conducted. Adapted from Hansen et al. [61]. (TIF) [file pone.0050971.s002.tif]

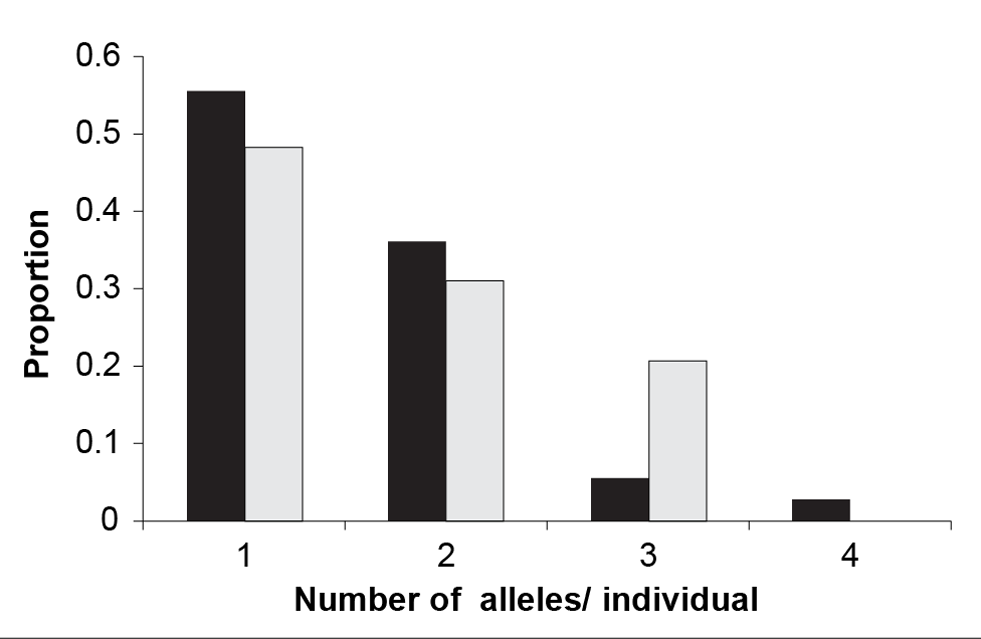

Supplement: Figure S2 — Frequency distribution of ELA- DQA copy number. DQA copy number frequency distribution in individuals from Etosha (black) versus Kruger (gray). One to four alleles were observed in each individual. Contingency goodness-of-fit analyses revealed no significant difference between population frequency distributions (χ2 = 5.578, p = 0.1341). (TIF) [file pone.0050971.s003.tif]

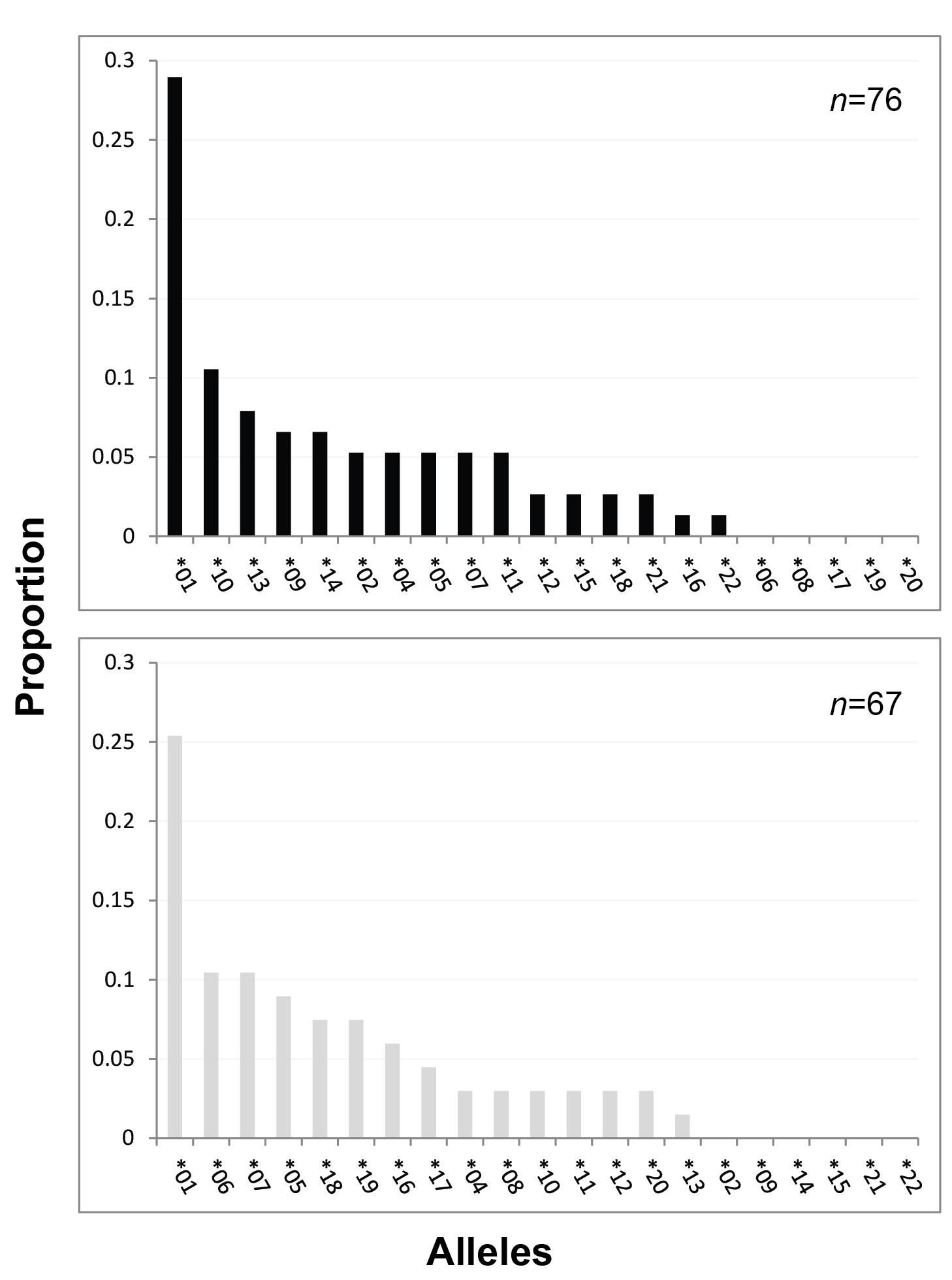

Supplement: Figure S3 — ELA- DQA allele frequency distributions, including alleles from multi-locus genotypes. Alleles are presented in descending order and by population, with Etosha in black and Kruger in gray. (TIF) [file pone.0050971.s004.tif]
