# Supplementary material for: Unraveling the Effects of Selection and Demography on Immune Gene Variation in Free-Ranging Plains Zebra (Equus quagga) Populations
Source: PLoS One. 2012 Dec 14;7(12):e50971. doi: 10.1371/journal.pone.0050971 (PMC3522668; doi:10.1371/journal.pone.0050971)
Supplement: Table S1 — Populations sampled and individuals successfully genotyped at each locus. Loci investigated include microsatellites (μsats), β-Fibrinogen intron 7 (β-Fibr), ELA-DRA exon 2 (DRA), and ELA-DQA exon 2 (DQA). The number of confirmed alleles found in each population is reported, with that for μsats reported as the mean (standard error) of 15 loci. Over both populations, typing success ranged from 41–71% per locus. (DOC) [file pone.0050971.s005.doc]

**Table S1. Populations sampled and individuals successfully genotyped at each locus**

Loci investigated include microsatellites (µsats), *β-Fibrinogen* intron 7 (*β-Fibr*), ELA-*DRA* exon 2 (*DRA*), and ELA-*DQA* exon 2 (*DQA*). The number of confirmed alleles found in each population is reported, with that for µsats reported as the mean (standard error) of 15 loci. Over both populations, typing success ranged from 41-71% per locus.

| **Population** | **% PCR success**  **(µsats/ *β-Fibr*/ *DRA*/ *DQA*)** | **No. samples typed**  **(µsats/ *β-Fibr*/ *DRA*/ *DQA*)** | **No. of Alleles** | | | |
| --- | --- | --- | --- | --- | --- | --- |
|  |  |  | µsats | *β-Fibr* | *DRA* | *DQA* |
| Etosha | 100/ 44/ 86/ 42 | 84/ 37/ 72/ 36 | 9.0 (0.9) | 8 | 8 | 16 |
| Kruger | 43/ 27/ 35/ 34 | 38/ 24/ 31/ 30 | 7.1 (0.7) | 6 | 8 | 15 |
