# Supplementary material for: Unraveling the Effects of Selection and Demography on Immune Gene Variation in Free-Ranging Plains Zebra (Equus quagga) Populations
Source: PLoS One. 2012 Dec 14;7(12):e50971. doi: 10.1371/journal.pone.0050971 (PMC3522668; doi:10.1371/journal.pone.0050971)
Supplement: Table S2 — Description of microsatellite loci. Includes total number of alleles, observed size range in base pairs (bp), fluorescent label used for genotyping analyses, annealing temperature used in polymerase chain reaction (PCR), and the reference study in which it was initially discovered in E. callabus. (DOC) [file pone.0050971.s006.doc]

**Table S2**. **Description of microsatellite loci**

Includes total number of alleles, observed size range in base pairs (bp), fluorescent label used for genotyping analyses, annealing temperature used in polymerase chain reaction (PCR), and the reference study in which it was initially discovered in *E. callabus*.

| **Locus** | **No. of Alleles** | **Range (bp)** | **Flourescent Label** | **PCR Annealing Temp. (°C)** | **Reference** |
| --- | --- | --- | --- | --- | --- |
| Aht21 | 10 | 196-214 | 6-FAM | TD* |  |
| Asb23 | 19 | 150-199 | NED | 60 |  |
| Cor014 | 14 | 134-165 | HEX | TD* |  |
| Hmb1 | 9 | 90-127 | PET | TD* |  |
| Hms7 | 9 | 161-184 | NED | TD* |  |
| Htg7 | 15 | 122-153 | 6-FAM | TD* |  |
| Htg9 | 12 | 114-140 | PET | 55 |  |
| Htg14 | 7 | 135-149 | HEX | 60 |  |
| Htg15 | 5 | 129-142 | HEX | TD* |  |
| Lex20 | 11 | 197-220 | 6-FAM | 60 |  |
| Lex33 | 3 | 163-177 | NED | 60 |  |
| Lex52 | 5 | 195-203 | 6-FAM | 60 |  |
| Ucdeq505 | 11 | 155-176 | 6-FAM | 55 |  |
| Um011 | 15 | 147-177 | HEX | 57 |  |
| Vhl47 | 5 | 123-133 | HEX | 60 |  |

*TD= “touch-down” thermocycling profile was used. This involved shifting the annealing temperature from 60°C to 50°C (see description in Methods).

**References**

1. Swinburne JE, Marti E, Breen M, Binns MM (1997) Characterization of twelve new horse microsatellite loci: AHT12-AHT23. Animal Genetics 28: 453-453.

2. Irvin Z, Giffard J, Brandon R, Breen M, Bell K (1998) Equine dinucleotide repeat polymorphisms at loci ASB 21, 23, 25 and 37-43. Animal Genetics 29: 67-67.

3. Hopman TJ, Han EB, Story MR, Schug MD, Aquadro CF, et al. (1999) Equine dinucleotide repeat loci COR001-COR020. Animal Genetics 30: 225-226.

4. Binns MM, Holmes NG, Holliman A, Scott AM (1995) The identification of polymorphic microsatellite loci in the horse and their use in thoroughbred parentage testing. British Veterinary Journal 151: 9-15.

5. Guerin G, Bertaud M, Amigues Y (1994) Characterization of 7 New Horse Microsatellites - Hms1, Hms2, Hms3, Hms5, Hms6, Hms7 and Hms8. Animal Genetics 25: 62.

6. Marklund S, Ellegren H, Eriksson S, Sandberg K, Andersson L (1994) Parentage testing and linkage analysis in the horse using a set of highly polymorphic microsatellites. Animal Genetics 25: 19-23.

7. Coogle L, Reid R, Bailey E (1996) Equine dinucleotide repeat loci LEX015-LEX024. Animal Genetics 27: 217-218.

8. Coogle L, Reid R, Bailey E (1996) Equine dinucleotide repeat loci from LEX025 to LEX033. Animal Genetics 27: 289-290.

9. Coogle L, Reid R, Bailey E (1997) Equine dinucleotide repeat loci LEX034-LEX048. Animal Genetics 28: 309-309.

10. Eggleston-Stott ML, DelValle A, Bautista M, Dileanis S, Wictum E, et al. (1997) Nine equine dinucleotide repeats at microsatellite loci UCDEQ136, UCDEQ405, UCDEQ412, UCDEQ425, UCDEQ437, UCDEQ467, UCDEQ487, UCDEQ502 and UCDEQ505. Animal Genetics 28: 370-371.

11. Meyer AH, Valberg SJ, Hillers KR, Schweitzer JK, Mickelson JR (1997) Sixteen new polymorphic equine microsatellites. Animal Genetics 28: 69-70.

12. van Haeringen WA, van de Goor LHP, van der Hout N, Lenstra JA (1998) Characterization of 24 equine microsatellite loci. Animal Genetics 29: 153-156.
