# Supplementary material for: Unraveling the Effects of Selection and Demography on Immune Gene Variation in Free-Ranging Plains Zebra (Equus quagga) Populations
Source: PLoS One. 2012 Dec 14;7(12):e50971. doi: 10.1371/journal.pone.0050971 (PMC3522668; doi:10.1371/journal.pone.0050971)
Supplement: Table S3 — Microsatellite diversity by locus and population. For each population, diversity by locus and total mean diversity are reported. Diversity is described in terms of number of alleles (A), observed heterozygosity (H O), and expected heterozygosity (H E). Sample sizes (N) are reported for each locus and population. Significant departures from Hardy-Weinberg equilibrium are indicated in boldface. (DOC) [file pone.0050971.s007.doc]

**Table S3. Microsatellite diversity by locus and population**

For each population, diversity by locus and total mean diversity are reported. Diversity is described in terms of number of alleles (*A*), observed heterozygosity (*H*_O_), and expected heterozygosity *(H*_E_). Sample sizes (*N*) are reported for each locus and population. Significant departures from Hardy-Weinberg equilibrium are indicated in boldface.

|  | **Etosha** | | | | |  | **Kruger** | | | | |
| --- | --- | --- | --- | --- | --- | --- | --- | --- | --- | --- | --- |
| **Locus** | *N* | *A* | *H_O_* | *H_E_* | *F_IS_* |  | *N* | *A* | *H_O_* | *H_E_* | *F_IS_* |
| Aht21 | 82 | 9 | 0.744 | 0.833 | 0.107 |  | 29 | 7 | 0.690 | 0.796 | 0.134 |
| Asb23 | 82 | 15 | 0.841 | 0.887 | 0.051 |  | 14 | 12 | 0.643 | 0.862 | 0.254 |
| Coro14 | 75 | 11 | 0.813 | 0.818 | 0.005 |  | 30 | 11 | 0.900 | 0.848 | -0.062 |
| Hmb1 | 79 | 8 | 0.658 | 0.675 | 0.025 |  | 37 | 6 | 0.811 | 0.763 | -0.063 |
| Hms7 | 79 | 8 | **0.595*** | 0.774 | 0.231 |  | 34 | 7 | 0.824 | 0.799 | -0.031 |
| Htg7 | 82 | 13 | 0.866 | 0.861 | -0.006 |  | 35 | 10 | 0.857 | 0.791 | -0.084 |
| Htg9 | 84 | 11 | 0.845 | 0.865 | 0.023 |  | 38 | 10 | 0.868 | 0.857 | -0.013 |
| Htg14 | 84 | 7 | 0.833 | 0.761 | -0.095 |  | 35 | 7 | 0.714 | 0.819 | 0.128 |
| Htg15 | 83 | 4 | **0.446*** | 0.651 | 0.315 |  | 34 | 5 | 0.912 | 0.689 | -0.323 |
| Lex20 | 81 | 11 | 0.765 | 0.821 | 0.068 |  | 29 | 7 | 0.724 | 0.829 | 0.126 |
| Lex33 | 78 | 3 | 0.615 | 0.605 | -0.018 |  | 11 | 3 | 0.455 | 0.376 | -0.209 |
| Lex52 | 75 | 5 | 0.693 | 0.740 | 0.064 |  | 34 | 4 | 0.765 | 0.659 | -0.160 |
| Ucdeq505 | 81 | 11 | 0.802 | 0.765 | -0.049 |  | 36 | 5 | 0.694 | 0.752 | 0.076 |
| Um011 | 80 | 14 | 0.875 | 0.865 | -0.012 |  | 34 | 9 | 0.706 | 0.730 | 0.033 |
| Vhl47 | 79 | 5 | 0.519 | 0.547 | 0.051 |  | 37 | 4 | 0.459 | 0.404 | -0.136 |
| ***MEAN*** | *80.27* | *9.00* | *0.76* | *0.76* | *0.05* |  | *31.13* | *7.13* | *0.73* | *0.73* | -0.02 |
| ***SE*** | *0.73* | *0.93* | *0.03* | *0.03* | *0.03* |  | *2.08* | *0.71* | *0.04* | *0.04* | 0.04 |

* *p* <0.01
