# Supplementary material for: Unraveling the Effects of Selection and Demography on Immune Gene Variation in Free-Ranging Plains Zebra (Equus quagga) Populations
Source: PLoS One. 2012 Dec 14;7(12):e50971. doi: 10.1371/journal.pone.0050971 (PMC3522668; doi:10.1371/journal.pone.0050971)
Supplement: Table S4 — Microsatellite null allele frequency and genotyping error rates. Null allele frequency (NAF) was estimated by population and locus. Total genotyping error rates, broken down by allelic dropout and false allele generation, were determined by paired genotyping of blood and fecal samples from individual zebra captures. All rates marked as “0” represent values of 0.000. (DOC) [file pone.0050971.s008.doc]

**Table S4.** **Microsatellite null allele frequency and genotyping error rates**

Null allele frequency (NAF) was estimated by population and locus. Total genotyping error rates, broken down by allelic dropout and false allele generation, were determined by paired genotyping of blood and fecal samples from individual zebra captures. All rates marked as “0” represent values of 0.000.

|  | ***NAF*** | | |  | ***Blood*** | | |  | ***Fecal*** | | |
| --- | --- | --- | --- | --- | --- | --- | --- | --- | --- | --- | --- |
| **Locus** | **Etosha** | **Kruger** | **All** |  | **Dropout** | **False Alleles** | **Total** |  | **Dropout** | **False Alleles** | **Total** |
| Aht21 | 0.044 | 0.051 | 0.055 |  | 0 | 0 | 0 |  | 0 | 0.048 | 0.048 |
| Asb23 | 0.021 | 0.058 | 0.038 |  | 0 | 0 | 0 |  | 0.048 | 0.024 | 0.071 |
| Cor014 | 0.007 | 0.000 | 0.006 |  | 0 | 0.024 | 0.024 |  | 0 | 0.071 | 0.071 |
| Hmb1 | 0.011 | 0.000 | 0.013 |  | 0 | 0 | 0 |  | 0 | 0 | 0 |
| Hms7 | 0.099 | 0.000 | 0.068 |  | 0 | 0 | 0 |  | 0 | 0 | 0 |
| Htg7 | 0.000 | 0.000 | 0.011 |  | 0 | 0 | 0 |  | 0 | 0.024 | 0.024 |
| Htg9 | 0.016 | 0.008 | 0.024 |  | 0 | 0 | 0 |  | 0 | 0.024 | 0.024 |
| Htg14 | 0.000 | 0.046 | 0.003 |  | 0 | 0 | 0 |  | 0.024 | 0 | 0.024 |
| Htg15 | 0.125 | 0.000 | 0.054 |  | 0 | 0.024 | 0.024 |  | 0 | 0.071 | 0.071 |
| Lex20 | 0.031 | 0.041 | 0.039 |  | 0 | 0 | 0 |  | 0 | 0 | 0 |
| Lex33 | 0.004 | 0.000 | 0.000 |  | 0 | 0 | 0 |  | 0 | 0 | 0 |
| Lex52 | 0.031 | 0.000 | 0.018 |  | 0 | 0 | 0 |  | 0 | 0.048 | 0.048 |
| Ucdeq505 | 0.000 | 0.016 | 0.001 |  | 0 | 0 | 0 |  | 0 | 0 | 0 |
| Um011 | 0.000 | 0.020 | 0.000 |  | 0 | 0 | 0 |  | 0 | 0 | 0 |
| Vhl47 | 0.020 | 0.000 | 0.010 |  | 0 | 0 | 0 |  | 0 | 0 | 0 |
| *Mean* | *0.027* | *0.016* | *0.023* |  | *0.000* | *0.003* | *0.003* |  | *0.005* | *0.021* | *0.025* |
