# Supplementary material for: Unraveling the Effects of Selection and Demography on Immune Gene Variation in Free-Ranging Plains Zebra (Equus quagga) Populations
Source: PLoS One. 2012 Dec 14;7(12):e50971. doi: 10.1371/journal.pone.0050971 (PMC3522668; doi:10.1371/journal.pone.0050971)
Supplement: Table S5 — Results from clustering analyses in STRUCTURE. Analyses were conducted over 10 runs of K = 1 to 5, combining all neutral data (13 microsatellites and the β-Fibr intron) and, separately, at each ELA locus. The mean posterior log probability (Mean L(K)), standard deviation (SD L(K)), mean difference between successive likelihood values of K (L′(K)), absolute value of the difference between successive values of L′(K) (|L″(K)|), and second order rate of change of the likelihood function with respect to K (ΔK) are reported. The best-fit models are highlighted in bold, and were selected based on mean posterior probabilities and following the approach described by Evanno et al. [83]. (DOC) [file pone.0050971.s009.doc]

**Table S5. Results from clustering analyses in STRUCTURE**

Analyses were conducted over 10 runs of *K*= 1 to 5, combining all neutral data (13 microsatellites and the *β-Fibr* intron) and, separately, at each ELA locus. The mean posterior log probability (Mean *L(K*)), standard deviation (SD *L(K*)), mean difference between successive likelihood values of *K* (*L'*(*K*)), absolute value of the difference between successive values of *L'*(*K*) (|*L''*(*K*)|), and second order rate of change of the likelihood function with respect to *K* (∆*K*) are reported. The best-fit models are highlighted in bold, and were selected based on mean posterior probabilities and following the approach described by Evanno *et al.* 2005.

|  | ***K*** | **Mean *L*(*K*)** | **SD *L(K*)** | ***L'(K*)** | **|*L''(K*)|** | **∆*K*** |
| --- | --- | --- | --- | --- | --- | --- |
| ***Neutral Data*** | 1 | -5692.49 | 0.28 | -- | -- | -- |
|  | **2** | **-5518.11** | **0.48** | **174.38** | **272.55** | **563.03** |
|  | 3 | -5616.28 | 11.71 | -98.17 | 135.64 | 11.58 |
|  | 4 | -5850.09 | 83.40 | -233.81 | 142.37 | 1.71 |
|  | 5 | -5941.53 | 87.99 | -91.44 | 91.44 | 1.04 |
| ***DRA*** | **1** | **-386.44** | **0.96** | -- | -- | -- |
|  | 2 | -390.25 | 8.38 | -3.81 | 13.08 | 1.56 |
|  | 3 | -407.14 | 15.22 | -16.89 | 20.42 | 1.34 |
|  | 4 | -403.61 | 8.29 | 3.53 | 28.53 | 3.44 |
|  | 5 | -428.61 | 17.76 | -25.00 | 25.00 | 1.41 |
| ***DQA*** | 1 | -305.29 | 1.12 | -- | -- | -- |
|  | **2** | **-270.84** | **2.74** | **34.45** | **63.54** | **23.18** |
|  | 3 | -299.93 | 10.50 | -29.09 | 15.39 | 1.47 |
|  | 4 | -313.63 | 12.51 | -13.70 | 9.74 | 0.78 |
|  | 5 | -317.59 | 19.74 | -3.96 | 3.96 | 0.20 |
